# USP7 inhibits the progression of nasopharyngeal carcinoma via promoting SPLUNC1-mediated M1 macrophage polarization through TRIM24

**DOI:** 10.1038/s41419-023-06368-w

**Published:** 2023-12-21

**Authors:** Huai Liu, Ling Tang, Sha Gong, Tengfei Xiao, Hongmin Yang, Wangning Gu, Hui Wang, Pan Chen

**Affiliations:** 1 Hunan Cancer Hospital and The Affiliated Cancer Hospital of Xiangya School of Medicine, Central South University, Changsha, 410013 Hunan Province P. R. China; 2 Key Laboratory of Translational Radiation Oncology, Hunan Province; Department of Radiation Oncology, Hunan Cancer Hospital and The Affiliated Cancer Hospital of Xiangya School of Medicine, Central South University, Changsha, 410013 Hunan Province P. R. China

**Keywords:** Oncogenes, Cancer

## Abstract

Reprogramming of macrophages toward an M1 phenotype is a novel strategy to induce anticancer immunity. However, the regulatory mechanisms of M1 macrophage polarization and its functional roles in nasopharyngeal carcinoma (NPC) progression need to be further explored. Here we found that SPLUNC1 was highly expressed and responsible for M1 macrophage polarization. JAK/STATs pathway activation was involved in SPLUNC1-mediated M1 macrophage polarization. Importantly, regulation of SPLUNC1 in macrophages affected CM-mediated influence on NPC cell proliferation and migration. Mechanistically, USP7 deubiquitinated and stabilized TRIM24, which promoted SPLUNC1 expression via recruitment of STAT3 in M1 macrophages. Depletion of TRIM24 inhibited M1 macrophage polarization, which facilitated NPC cell growth and migration. However, over-expression of USP7 exhibited the opposite results and counteracted the tumorigenic effect of TRIM24 silencing. Finally, the growth and metastasis of NPC cells in vivo were repressed by USP7-induced M1 macrophage polarization via modulating TRIM24/SPLUNC1 axis. USP7 delayed NPC progression via promoting macrophage polarization toward M1 through regulating TRIM24/SPLUNC1 pathway, providing evidence for the development of effective antitumor immunotherapies for NPC.

## Introduction

Nasopharyngeal carcinoma (NPC) is a common malignancy occurred in the nasopharynx, which occupies approximately 78% of all head and neck tumors [1]. There is a higher incident rate of NPC in southern China and Southeast Asia [2]. Currently, the primary therapy for NPC is surgical excision followed by radiotherapy and chemotherapy, however, the prognosis for NPC patients with metastasis is not ideal [3]. Thus, elucidation of the mechanisms of NPC cell metastasis and identification of novel interventions are urgently needed.

Macrophages, also known as tumor-associated macrophages in cancers, which commonly exhibit M2 phenotype and confer tumor development [4]. While M1 phenotype macrophages produce a pro-inflammatory Th1 immunoreaction and exert tumor killing activity [5]. Tumor-associated macrophages exist in the tumor microenvironment of NPC and have been reported to associate with the outcome of NPC patients [6]. However, the regulatory mechanism of macrophage polarization during NPC progression has not been clarified.

Short palate, lung and nasal epithelium clone 1 (SPLUNC1) is a member of PLUNC family expressed in nasopharynx, possessing host defense property [7]. SPLUNC1 has been shown to protect against Gram-negative bacteria-induced respiratory inflammation [8]. A previous study indicated that SPLUNC1 exerted tumor-suppressor effect on Epstein-Barr virus-induced inflammatory response in NPC cells [9]. Zhang et al. reported that SPLUNC1 expression was reduced in NPC samples, and positive expression of SPLUNC1 in NPC predicted a better prognosis [10]. So far, whether SPLUNC1 can delay NPC progression via modulating macrophage polarization remains obscure. A published study has revealed that tripartite motif-containing 24 (TRIM24) deficiency promoted M2 macrophage polarization, which impaired antitumor immunity [11]. Moreover, TRIM24 has been shown to drive tumorigenesis via recruiting signal transducer and activator of transcription 3 (STAT3) in glioblastoma [12]. Interestingly, through AnimalTFDB prediction online (http://bioinfo.life.hust.edu.cn/AnimalTFDB/#!/), we found that both TRIM24 and STAT3 were predicted to possess binding sites in the promoter of SPLUNC1. In this context, we speculated that TRIM24 might recruit STAT3 to regulate SPLUNC1 expression, and thereby modulating macrophage polarization in NPC.

Ubiquitin-specific processing protease 7 (USP7) is a pivotal deubiquitinating enzyme [13]. USP7 has been documented to play crucial roles in tumor development via modulating the stability and function of specific proteins [14, 15]. More recently, Palazón-Riquelme et al. reported that USP7 was responsible for NLRP3 inflammasome activation [16]. Nevertheless, the biological functions of USP7 in NPC are still largely unknown. As analyzed by bioinformatics, USP7 has the potential to deubiquitylate and stabilize TRIM24. However, whether USP7 could affect NPC progression via deubiquitylation and stabilization of TRIM24, and subsequently regulate SPLUNC1-mediated M1 macrophage polarization remains elusive. In this study, we demonstrated that up-regulation of SPLUNC1 was essential for M1 macrophage polarization. SPLUNC1 modulated the anti-tumor effect of M1 macrophages. Moreover, TRIM24 enhanced SPLUNC1 expression via recruitment of STAT3. USP7 stabilized TRIM24 to up-regulate SPLUNC1, which repressed NPC cell growth and metastasis. Our observations uncover the mechanisms of reprogramming of macrophages in NPC and identify targeting USP7/TRIM24/SPLUNC1 axis as an effective immunotherapy for NPC.

## Materials and methods

### Cell culture and treatment

Human acute monocytic leukemia cell line THP-1 cells, NPC cell lines (NPC/HK-1 and CNE3) were cultured in RMPI-1640 containing 10% fetal bovine serum (FBS, Gibco, USA) at 37 °C with 5% CO_2_. The cell lines were authenticated by STR DNA profiling analysis and tested for mycoplasma contamination. The peripheral blood mononuclear cells (PBMCs) were separated from the human peripheral blood collected from healthy donors with informed consent using a Histopaque®-1077 density gradient (Sigma-Aldrich, USA) according to a previous study [17]. The study was approved by the Ethical Committee of Hunan Cancer Hospital and the Affiliated Cancer Hospital of Xiangya School of Medicine, Central South University.

For the induction of macrophage (Mφ) differentiation, CD14^+^ monocytes were separated from PBMCs using the classical monocyte isolation kit (Miltenyi Biotech, Germany), followed by stimulation with 25 ng/mL M-CSF (R&D Systems, USA) in culture medium for 7 days. THP-1 cells were first differentiated into Mφs with 50 ng/mL phorbol 12-myristate 13-acetate (PMA, Sigma-Aldrich) for 24 h. To induce M1 polarization, PBMC or THP-1-derived Mφs were subjected to 15 ng/mL lipopolysaccharide (LPS, Sigma-Aldrich) and 20 ng/mL interferon-γ (IFN-γ, Sigma-Aldrich), while M2 polarization was induced by treatment with 20 ng/mL IL-13 (Sigma-Aldrich) and 20 ng/mL IL-4 (Sigma-Aldrich).

### Cell transfection

For gene knockdown, the short hairpin RNA (shRNA) targeting SPLUNC1 (shSPLUNC1), TRIM24 (shTRIM24), USP7 (shUSP7), STAT1 (shSTAT1), STAT3 (shSTAT3), STAT6 (shSTAT6), and negative control shRNA (shNC) were provided by GenePharma (Shanghai, China). shSPLUNC1 sequences: 5’-CACCGAAATCTTAGCTGTGAGAGATCGAAAT

CTCTCACAGCTAAGATTTC-3’ shTRIM24 sequences: 5’-CACCAAAGCAGGTGGAACAG

GATATTAAACGAATTTAATATCCTGTTCCACCTGC-3’; shUSP7 sequences: 5’-CACCGCCTGGATTTGTGGTTACGTTACGAATAACGTAACCACAAATCCAGG-3’; shSTAT1 sequences: 5’-CACCGAACAGAAATACACCTACGAACGAATTCGTAGGTGTATTTCTGTTC-3’; shSTAT3 sequences: 5’-CACCGCTGACCAACAATCCCAAGAACGAATTCTTGGGATTGTTGGTCAGC-3’; shSTAT6 sequences: 5’-CACCGCAGGAACATACAGACACATTCGAAAATGTGTCTGTATGTTCCTGC-3’; For gene overexpression, the overexpression plasmids for USP7, SPLUNC1 and their empty vector (EV) were constructed by GenePharma. THP-1-derived Mφs were transfected with the above segments using Lipofectamine 2000 (Thermo Fisher, USA).

### Real-time quantitative PCR (RT-qPCR)

Total RNA was obtained using TRIzol reagent (Thermo Fisher), and then reverse transcribed into cDNA using the Prime Script™ RT reagent kit (TAKARA, Japan). Real-time PCR was carried out using the SYBR Green Mix (Vazyme, Nanjing, China). The mRNA expression levels normalized to GAPDH were calculated according to the 2^-ΔΔCT^ method. The primer sequences are shown in Table 1.

**Table 1:** Oligonucleotide primer sets for qPCR.

Name	Sequence (5’¬3’)	Length
CD86 F	CTTTGCTTCTCTGCTGCTGT	20
CD86 R	GGCCATCACAAAGAGAATGTTAC	23
SPLUNC1 F	CCCATTCAAGGTCTTCTGGA	20
SPLUNC1 R	CTGTAGTCCGTGGATCAGCA	20
IL-1β F	CCACAGACCTTCCAGGAGAATG	22
IL-1β R	GTGCAGTTCAGTGATCGTACAGG	23
IL-12 F	GCATTCTTCACCTGCTCCAC	20
IL-12 R	ATCCTCTCCTGTTGGCACTG	20
iNOS F	GTGGTGACAAGCACATTTGG	20
iNOS R	GTCATGAGCAAAGGCACAGA	20
IL-10 F	CTGGTTCTGGGTGATGTTGAC	21
IL-10 R	CTCGCTTCGGCAGCACA	17
Arg-1 F	CAGAAGAATGGAAGAGTCAG	20
Arg-1 R	CAGATATGCAGGGAGTCACC	20
Mrc-1 F	CTCTGTTCAGCTATTGGACGC	21
Mrc-1 R	CGGAATTTCTGGGATTCAGCTTC	23
GAPDH F	ATCCACGGGAGAGCGACAT	19
GAPDH R	CAGCTGCTTGTAAAGTGGAC	20

### Western blotting

Total protein samples were extracted with RIPA buffer (Beyotime, Haimen, China), and quantified using the protein assay reagent (Beyotime). Subsequently, sodium-dodecyl-sulfate polyacrylamide gel electrophoresis was performed, followed by transferring onto polyvinylidene fluoride membranes. Following blocking in 5% BSA for 2 h, the membranes were then probed with primary antibodies against CD86 (1:500, A19026, Abclonal, Wuhan, China), CD163 (1:500, A8383, Abclonal), CD206 (1:1000, ab64693, Abcam), SPLUNC1 (1:200, 10413-1-AP, Proteintech, Wuhan, China), STAT3 (1:500, A19566, Abclonal), p-STAT3-Tyr705 (1:500, AP0070, Abclonal), STAT6 (1:500, A19120, Abclonal), p-STAT6-Tyr641 (1:500, AP1390, Abclonal), JAK1 (1:500, A18323, Abclonal), p-JAK1-Tyr1034/1035 (1:1000, 74129, CST, USA), JAK2 (1:500, A19629, Abclonal), p-JAK2-Tyr1007/1008 (1:500, AP0531, Abclonal), TRIM24 (1:2000, 14208-1-AP, Proteintech), USP7 (1:500, A3448, Abclonal), β-actin (1:5000, AC006, Abclonal) at 4 °C overnight. Membranes were then reacted with secondary antibody. Immunoreactive bands were detected using the Super ECL Detection Reagent (Yeasen, Shanghai, China).

### Flow cytometry

The expression of macrophage markers was analyzed by flow cytometry. Single cell suspension of macrophages was prepared and stained with CD86 PE (12-0869-42, EBiosciences, USA), CD206 APC (17-2069-42, EBiosciences), CD11b FITC (11-0113-42, EBiosciences) on ice away from light. After washing with PBS, the macrophages were detected on a flow cytometer (Thermo Fisher).

### Immunohistochemical (IHC) staining

NPC tissue chip was purchased from Auragene Bioscience (Changsha, Hunan), which was used for IHC staining. The xenograft tumor tissues were paraffin-embedded and sliced into 4-μm slices. After deparaffinization in xylene and dehydration, the peroxidase in slices was blocked with 3.0% hydrogen peroxide. Subsequently, the slides were probed with primary antibodies against SPLUNC1 (1:50, 10413-1-AP, Proteintech), CD86 (1:100, ab220188, Abcam), Ki67 (1:50, ab16667, Abcam), TRIM24 (1:200, ab70560, Abcam), and USP7 (1:100, ab264422, Abcam) overnight at 4 °C, followed by incubation with HRP-conjugated goat anti-rabbit IgG. All slices were reacted with DAB, counterstained with Meyer’s hematoxylin, and photographed under a microscope. The percentage of positive staining cells was quantified using Image J software.

### Immunofuorescence (IF) staining

Immunofuorescence staining was performed using an anti-CD86 antibody (1:100 dilution, ab239075, Abcam) and an anti-SPLUNC1 antibody (1:100 dilution, 10413-1-AP, Proteintech). Images were captured after nuclear staining with DAPI under a fluorescence microscope (Leica, Germany).

### CCK-8 assay

Cell proliferation was measured using the CCK-8 reagent (Yeasen, Shanghai, China). In brief, cells plated in 96-well plates (2000 cells per well) were reacted with 10 μL CCK-8 solution (5 mg/mL) for 4 h. After dissolving in DMSO, the absorbance at 450 nm was recorded on a microplate reader (Tecan, Switzerland).

### Colony formation

NPC cells received various treatments were planted into 6-well plates (200 cells per well) and cultured for 14 days. Fixation in 4% paraformaldehyde and then staining with 0.1% crystal violet was performed on colonies. The images were captured by a camera and the colonies were manually counted.

### Transwell assay

Cell migratory ability was assessed using the transwell chambers (8-mm pore size, Corning, USA). Briefly, NPC cells were resuspended in serum-free medium and added into the upper inserts. While 600 µL complete medium with 10% FBS was added to the lower chamber. After maintenance at 37 °C for 24 h, the migrated cells on the lower chambers were subjected to 4% paraformaldehyde fixation and 0.1% crystal violet staining. Under a light microscope, the stained cells were imaged and counted.

### Enzyme-linked immunosorbent assay (ELISA)

The supernatants of NPC cells were collected, and levels of IL-10, IL-4, TGF-β, TNF-γ, and TNF-α were determined using the commercial ELISA Kits purchased from USCN (Wuhan, China) according to the manufacturer’s instructions.

### Dual-luciferase reporter assay

The wild-type (WT) SPLUNC1 promoter sequences contained the conserved SPLUNC1 binding sites or its mutant (MUT) sequences were sub-cloned into the luciferase reporter vector pGL3 (Promega, USA). The THP-1-derived Mφs were transfected with pGL3-SPLUNC1 plasmid together with sh- SPLUNC1, sh-TRIM24, shSTAT3, sh-TRIM24+shSTAT3, or shNC. Firefly luciferase activity normalized to Renilla luciferase activity was detected using the Dual Luciferase Reporter Gene Assay Kit (Yeasen) at 24 h after transfection.

### Chromatin immunoprecipitation (ChIP)

ChIP assay was performed to validate the binding of TRIM24/STAT3 to SPLUNC1 promoter using the High-Sensitivity ChIP Kit (Abcam, UK). Briefly, the macrophages were treated with 1% formaldehyde for cross-linking and then subjected to ultrasound for the generation of DNA fragments. After centrifugation at 4 °C, 13000 rpm, the supernatant was co-immunoprecipitated with anti-SPLUNC1 (10413-1-AP, Proteintech) or anti-IgG antibody (30000-0-AP, Proteintech) overnight at 4 °C. Finally, the protein/DNA complexes were purified and detected by RT-qPCR.

### Co-immunoprecipitation (Co-IP)

For Co-IP, the macrophages were treated with the Pierce IP lysis buffer (Thermo Fisher) containing the protease inhibitor cocktail (Roche, Switzerland) to prepare cell lysates. Then, IP was conducted using anti-USP7 (ab264422, Abcam), anti-TRIM24 (ab70560, Abcam), anti-STAT3 (A19566, Abclonal), anti-STAT1 (ab234400, Abcam), anti-STAT6 (ab32108, Abcam), anti-Ubiquitin (ab179434, Abcam) or control IgG (30000-0-AP, Proteintech) that was pre-conjugated to protein A/G PLUS-Agarose overnight at 4 °C. The immunoprecipitated proteins were eluted and detected by Western blotting. P5091(HY-15667, MedChemExpress) or GNE6640 (HY-112937, MedChemExpress) were used to impede the USP7.

### Protein stability assay

The macrophages were treated with CHX (5 μg/mL, Sigma-Aldrich) for 0, 15, 30, 60, 120, and 240 min. Cells were collected and lysed at different time points, followed by western blot analysis. The results were normalized and quantified using Quantity One Software (Bio-Rad).

### Animal model

A total of 1 × 10^6^ NPC/NK-1 and CNE3 cells were suspended in 100 µL PBS or conditioned medium (CM) from THP-1-derived Mφs that were transfected with shTRIM24, USP7 over-expression plasmid or shTRIM24 + USP7 over-expression plasmid. Subsequently, the cell suspension was mixed with 250 µL ice-cold matrigel (BD Biosciences, USA), which were subcutaneously injected into the BALB/c nude mice (male, 5 weeks old) obtained from Slac Jingda Laboratory Animal Co., Ltd (Hunan, China). The mice were divided into 5 experimental groups (*n* = 4 per group) via block pseudo-randomization: Mφ CM (-), Mφ CM (+), shTRIM24 Mφ CM ( + ), USP7 Mφ CM (+), shTRIM24 + USP7 Mφ CM (+). Tumor volume was monitored every five days using the formula: (length × width^2^)/2. The investigators were blinded to grouping assignment. Mice were euthanized and the tumor tissues were collected and weighed 30 days after injection.

The in vivo metastatic ability was assessed in nude mice by tail vein injection with 2 × 10^6^ NPC cells (NPC/NK-1 and CNE3) resuspended in 100 µL PBS or CM in combination with 250 µL matrigel as described above. Two months after injection, all mice were euthanized to observe metastatic nodules in lung tissues. The collected lung tissue samples were received fixation in formalin, embedding in paraffin, and slicing into 5-μm sections, followed by staining using the Hematoxylin-Eosin (HE) Staining Kit for histological examination. All animal protocols were approved by the Ethics Committee of Hunan Cancer Hospital and the Affiliated Cancer Hospital of Xiangya School of Medicine, Central South University.

### Statistical analysis

Sample size calculation was not performed, while sample sizes were based on previous studies using similar analysis of xenograft model [18, 19]. The normality of data was analyzed by the Shapiro-Wilk test and all data are normally distributed (*P* > 0.05). All the results are presented as mean ± standard deviation (SD) from three independent experiments. One-way analysis of variance (ANOVA) followed by Tukey’s post-hoc test or Student’s *t*-test was adopted for statistical analysis using GraphPad Prism 7 software. The variance was similar between the groups and was statistically compared. Significance was defined as *p* < 0.05.

## Results

### SPLUNC1 was up-regulated during M1 macrophage polarization

**Fig. 1:**
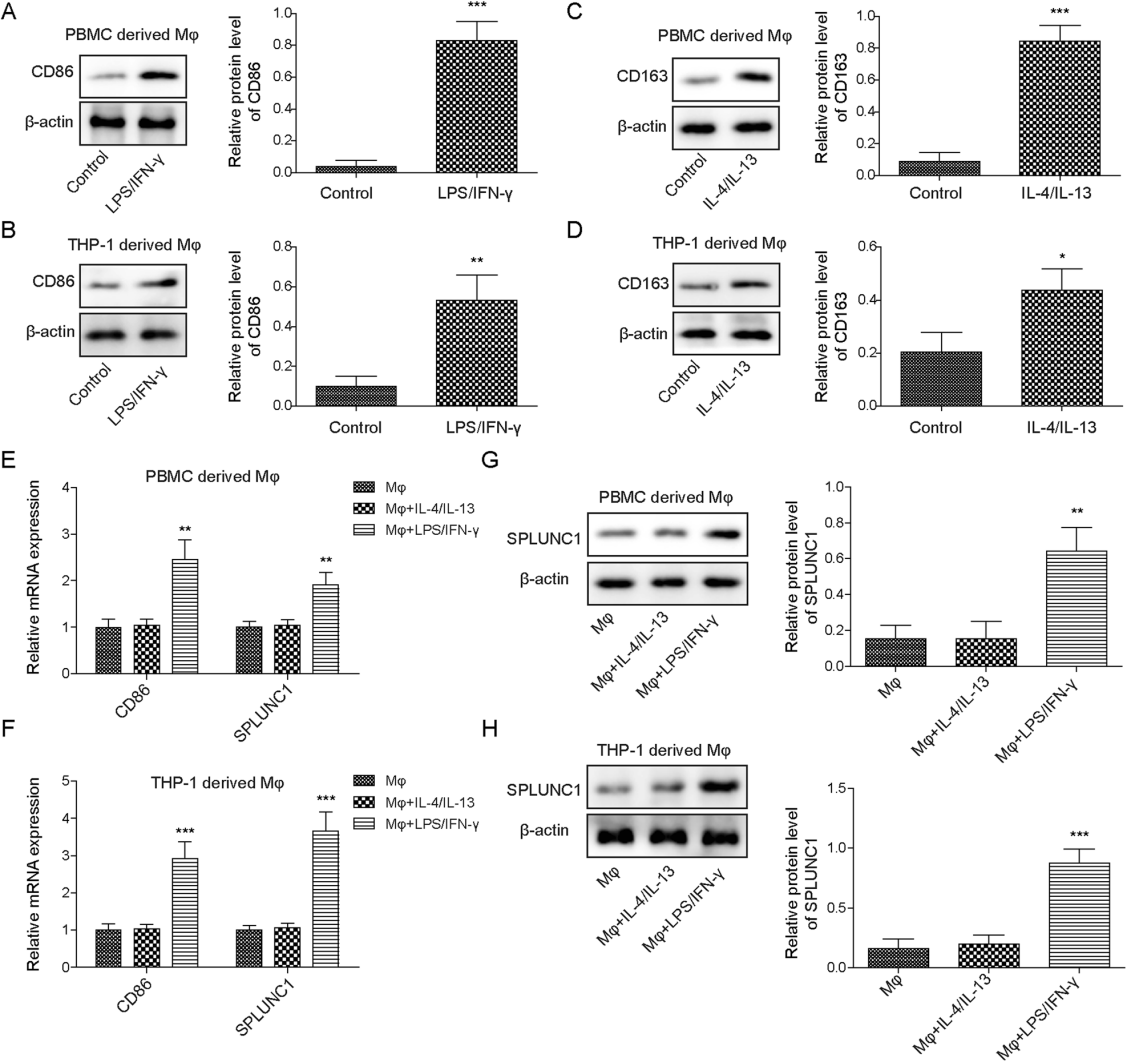
Up-regulation of SPLUNC1 in M1 macrophages. M1 macrophages PBMC- or THP-1-derived Mφs were stimulated by 15 ng/mL LPS + 20 ng/mL IFN-γ for M1 polarization, and triggered by 20 ng/mL IL-4 + 20 ng/mL IL-13 for M2 polarization. The protein levels of M1 macrophage marker CD86 (**A**, **B**) and M2 macrophage marker CD163 (**C**, **D**) in PBMC- or THP-1-derived Mφs macrophages were assessed by Western blotting. The mRNA expression of CD86 and SPLUNC1 in PMBC derived Mφ (**E**) and THP-1 derived Mφ (**F**) was detected by RT-qPCR. **G**, **H** SPLUNC1 protein expression was evaluated by Western blotting. *p < 0.05, **p < 0.01, ***p < 0.001. For A-D, Student’s *t*-test was performed. For (**E**–**H**) one-way ANOVA followed by Tukey’s multiple comparison test was performed.

To investigate the differential expression of SPLUNC1 during macrophage polarization, PBMCs- or THP-1-derived Mφs were stimulated with LPS/IFN-γ or IL-4/IL-13 for M1 or M2 macrophage polarization. The identification of M1 and M2 macrophages was performed by determining macrophage markers, CD86 (M1) and CD163/CD206 (M2). As illustrated in Fig. 1A–D, CD86 or CD163 was up-regulated after respective treatment, indicating the polarization of M1 or M2 macrophages. Moreover, the mRNA expression of SPLUNC1 and CD86 in PBMCs- and THP-1-derived Mφs was enhanced by LPS/IFN-γ, but not IL-4/IL-13 stimulation (Fig. 1E, F). Similarly, LPS/IFN-γ, but not IL-4/IL-13 treatment resulted in a remarkable increase in SPLUNC1 protein level (Fig. 1G and H). Notably, treatment with LPS alone did not affect the expression of SPLUNC1, CD86, and CD206 (Supplementary Fig. 1A). IFN-γ single treatment slightly increased CD86 and SPLUNC1 expression, and reduced CD206 expression. On the contrary, IL-4 or IL-13 single treatment slightly decreased CD86 and SPLUNC1 expression, and enhanced CD206 expression (Supplementary Fig. 1A). These changes were more significant in LPS + IFN-γ or IL-4 + IL-13 co-treatment groups (Supplementary Fig. 1A). Moreover, CD86 and SPLUNC1 were time dependently up-regulated by LPS + IFN-γ, but time dependently down-regulated by IL-4 + IL-13 (Supplementary Fig. 1B). CD206 expression was reduced by LPS + IFN-γ, while enhanced by IL-4 + IL-13 in a time dependent manner (Supplementary Fig. 1B). These data supported that SPLUNC1 expression was elevated during M1 macrophage polarization.

### SPLUNC1 was responsible for M1 macrophage polarization

**Fig. 2:**
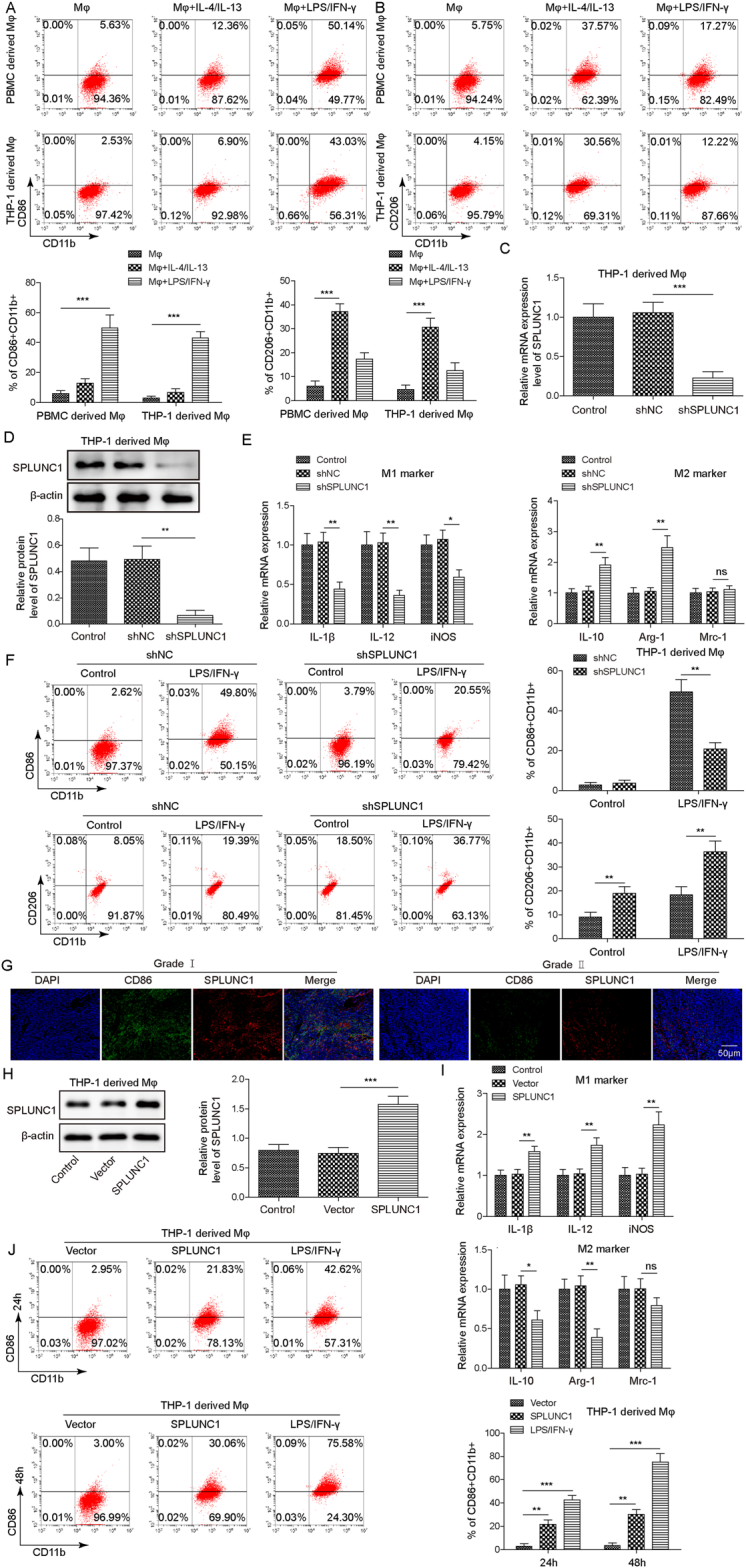
SPLUNC1 was essential for M1 macrophage polarization. **A**, **B** Flow cytometry analysis of percentage of M1 (CD86^+^ CD11b^+^ ) and M2 (CD206^+^ CD11b^+^ ) macrophages after treatment with 15 ng/mL LPS + 20 ng/mL IFN-γ or 20 ng/mL IL-4 + 20 ng/mL IL-13. **C**, **D** RT-qPCR and Western blotting validated the silencing efficiency of SPLUNC1 in THP-1-derived Mφs after transfection with shSPLUNC1 for 48 h. **E** The mRNA expression of M1 (IL-1β, IL-12, iNOS) and M2 (IL-10, Arg-1, Mrc-1) markers in THP-1-derived Mφs was measured by RT-qPCR. **F** Percentages of M1 (CD86^+^ CD11b^+^ ) macrophages and M2 (CD206^+ ^CD11b^+^ ) macrophages in THP-1-derived Mφs were analyzed by flow cytometry. **G** The expression of CD86 and SPLUNC1 in NPC tissue was assessed by immunofluorescence staining (magnification, 200×). Scale bar = 50 μm. **H** The overexpression efficiency of SPLUNC1 in THP-1-derived Mφs after transfection with oe-SPLUNC1 plasmid for 48 h was confirmed by Western blotting. **I** RT-qPCR determined the mRNA levels of M1 and M2 markers in THP-1-derived Mφs. SPLUNC1 group represents THP-1-derived Mφs transfected with SPLUNC1 overexpression plasmid. **J** CD86^+^ CD11b^+ ^M1 macrophage percentage in THP-1-derived Mφs was detected by flow cytometry. *p < 0.05, **p < 0.01, ***p < 0.001. One-way ANOVA followed by Tukey’s multiple comparison test was performed.

Next, the biological function of SPLUNC1 in the phenotypical change of M1 macrophages was examined. CD86^+^ CD11b^+^ M1 macrophage proportion was strikingly increased by LPS/IFN-γ stimulation, while CD206^+^ CD11b^+^ M2 macrophage proportion was raised by IL-4/IL-13 stimulation (Fig. 2A, B). After shSPLUNC1 transfection, the silencing efficiency in THP-1-derived Mφs was validated (Fig. 2C, D). Depletion of SPLUNC1 led to a decrease in expression of M1 markers (IL-1β, IL-12 and iNOS), while an elevation in expression of M2 markers (IL-10 and Arg-1) (Fig. 2E). However, Mrc-1 expression level was not affected by SPLUNC1 silencing (Fig. 2E). We further validated that SPLUNC1 knockdown reduced CD86^+^ CD11b^+ ^M1 macrophage percentage stimulated by LPS/IFN-γ (Fig. 2F). While CD206^+ ^CD11b^+^ M2 macrophage percentage was enhanced by SPLUNC1 knockdown, which was further strengthened in LPS/IFN-γ-stimulated cells (Fig. 2F). Furthermore, IF staining showed that the percentage of positive expression of CD86 and SPUNC1 was lower in the NPC tissues with higher grade malignancy (Fig. 2G). To further explore the regulatory function of SPLUNC1, we transfected SPLUNC1 plasmid to overexpress SPLUNC1 in THP-1-derived Mφs (Fig. 2H). As compared with the empty vector (EV) group, M1 markers (IL-1β, IL-12 and iNOS) levels were increased, but M2 markers (IL-10 and Arg-1) levels were decreased in SPLUNC1-overexpressed group (Fig. 2I). Whereas M2 marker Mrc-1 expression was not changed after SPLUNC1 overexpression (Fig. 2I). Consistently, the M1 macrophage proportion was enhanced by SPLUNC1 overexpression with the increase of induction time (Fig. 2J). In addition, treatment with CM from Mφs remarkably reduced IL-10, IL-4 and TGF-β levels in NPC cells, which was reversed by SPLUNC1 deficiency, but intensified by SPLUNC1 overexpression (Supplementary Fig. 1C). Conversely, NF-γ and TNF-α levels in NPC cells were enhanced by treatment with CM from Mφs, which was abolished by CM from SPLUNC1-silenced Mφs, but strengthened by CM from SPLUNC1-overexpressed Mφs (Supplementary Fig. 1D). Collectively, SPLUNC1 can promote the phenotypical change of M1 macrophages.

### SPLUNC1 contributed to M1 macrophage polarization via JAK/STATs pathway

**Fig. 3:**
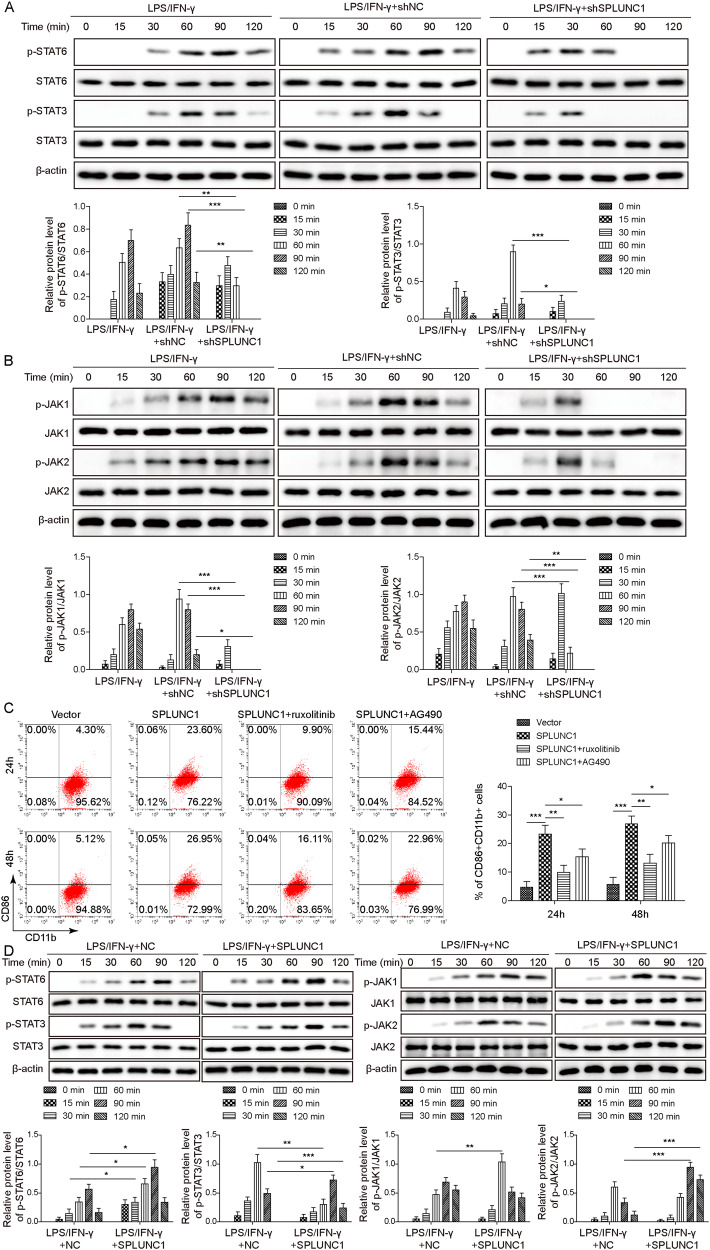
Activation of JAK/STATs pathway was involved in SPLUNC1-induced M1 macrophage polarization. **A** After stimulation with LPS/IFN-γ for 0, 15, 30, 60, 90, 120 min, the protein levels of p-STAT6, STAT6, p-STAT3, STAT3 in THP-1-derived Mφs that were transfected with shNC or shSPLUNC1 were assessed by Western blotting. **B** After stimulation with LPS/IFN-γ for 0, 15, 30, 60, 90, 120 min, the protein levels of p-JAK1, JAK1, p-JAK2, JAK2 in THP-1-derived Mφs that were transfected with shNC or shSPLUNC1 were assessed by Western blotting. **C** The percentage of M1 (CD86^+^ CD11b^+^ ) macrophages in THP-1-derived Mφs after overexpression of SPLUNC1 with or without treatment with JAK/STATs pathway inhibitors (AG490, Ruxolitinib) was detected by flow cytometry. **D** After stimulation with LPS/IFN-γ for 0, 15, 30, 60, 90, 120 min, the protein levels of p-JAK1, JAK1, p-JAK2, JAK2, p-STAT6, STAT6, p-STAT3, STAT3 in THP-1-derived Mφs transfected with vector or oe-SPLUNC1 were assessed by Western blotting. *p < 0.05, **p < 0.01, ***p < 0.001. One-way ANOVA followed by Tukey’s multiple comparison test was performed.

To clarify the mechanisms underlying SPLUNC1-induced M1 macrophage polarization, JAK2/STATs pathway was focused on. Figure 3A and B showed that the phosphorylation of STAT6, STAT3, JAK1, and JAK2 was triggered in THP-1-derived Mφs after stimulation with LPS/IFN-γ for 30 min, which was weakened at 120 min. However, depletion of SPLUNC1 remarkably abolished these changes. In addition, treatment with JAK/STATs pathway inhibitor Ruxolitinib or AG490 distinctly reversed SPLUNC1 overexpression-induced increased proportion of M1 macrophages (CD86^+ ^CD11b^+^ ) (Fig. 3C). Contrary to SPLUNC1 silencing, the phosphorylation of STAT6, STAT3, JAK1, and JAK2 in response to LPS/IFN-γ was obviously strengthened by SPLUNC1 over-expression (Fig. 3D). These data demonstrated that SPLUNC1 drove M1 macrophage polarization via activating the JAK/STATs pathway.

### SPLUNC1-induced M1 macrophage polarization repressed growth and migration of NPC cells

**Fig. 4:**
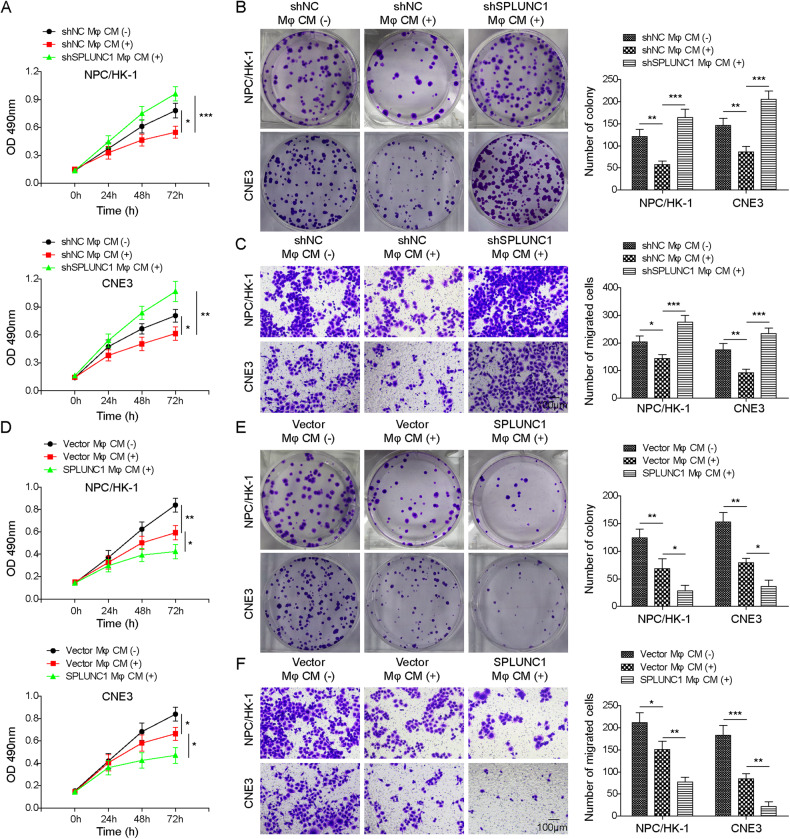
SPLUNC1-mediated M1 macrophage polarization restrained malignant capacities of NPC cells. NPC cells were administrated with CM collected from THP-1-derived Mφs transfected with shNC or shSPLUNC1. **A**, **B** The growth or NPC cells was evaluated by CCK-8 and colony formation assay. **C** Transwell assay for detection of migratory ability of NPC cells. Scale bar = 100 μm. NPC cells were administrated with CM from THP-1-derived Mφs transfected with vector or SPLUNC1 overexpression plasmid. **D**, **E** CCK-8 and colony formation assay for assessing NPC cell proliferation. **F** Migration of NPC cells was measured by Transwell assay. Scale bar = 100 μm *p < 0.05, **p < 0.01, ***p < 0.001. One-way ANOVA followed by Tukey’s multiple comparison test was performed.

To determine whether SPLUNC1-mediated M1 macrophage polarization played anti-tumor roles, NPC cells (NPC/HK-1 and CNE3) were added with CM from THP-1-derived Mφs. As compared with control cells, treatment with CM inhibited the growth of NPC cells, whereas this result was disrupted by knockdown of SPLUNC1 in THP-1-derived Mφs (Fig. 4A, B). The migration of NPC cells was repressed by CM administration, which was counteracted by SPLUNC1 silencing (Fig. 4C). On the contrary, administration with CM from SPLUNC1-overexpressed THP-1-derived Mφs further reinforced the inhibitory effect of CM from THP-1-derived Mφs on proliferation and migration of NPC cells (Fig. 4D–F). These findings suggested that SPLUNC1-induced M1 macrophage polarization inhibited NPC cell growth and metastasis in vitro.

### High expression of TRIM24 in M1 macrophages enhanced SPLUNC1 expression via recruitment of STAT3

**Fig. 5:**
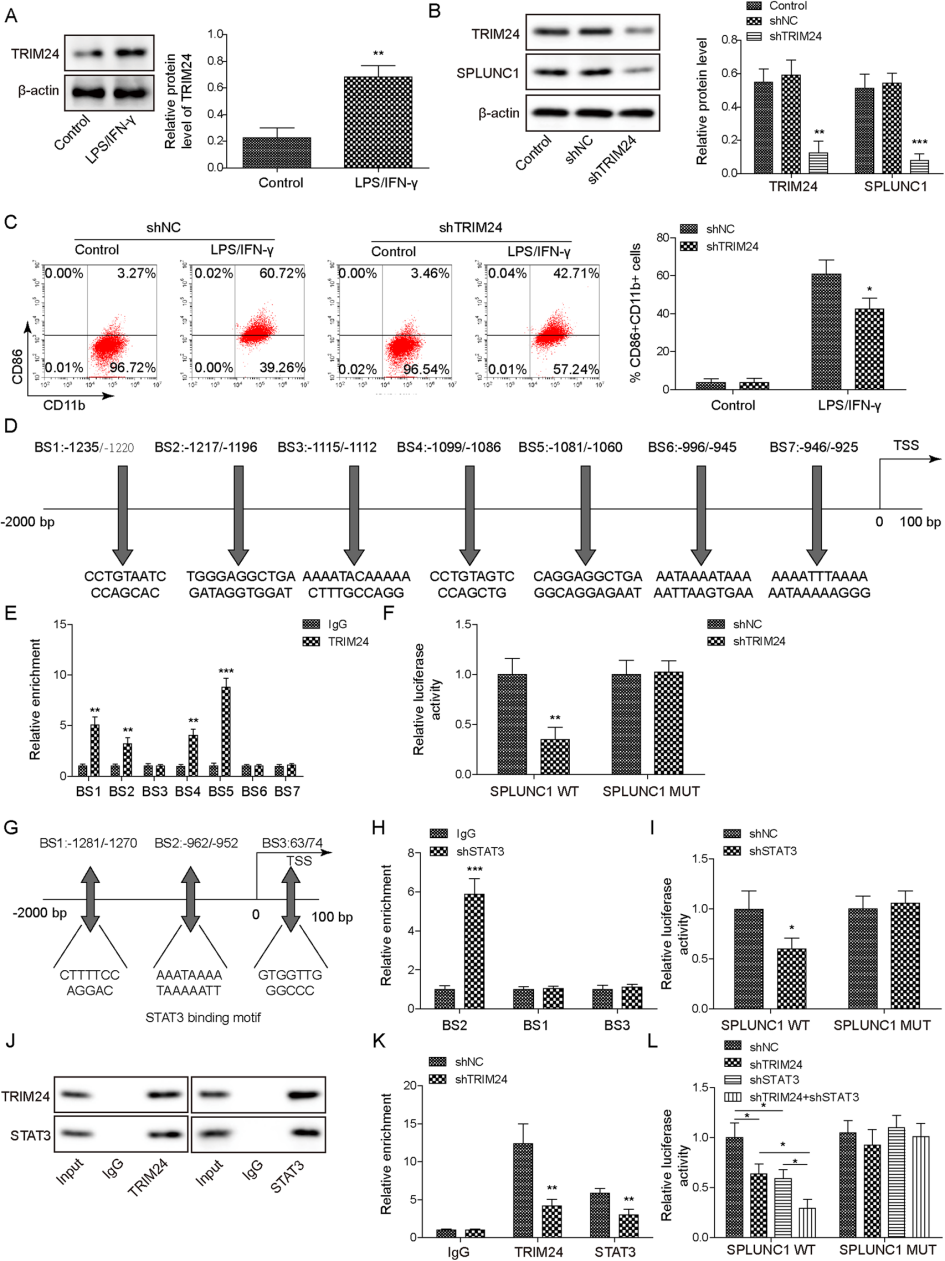
TRIM24 recruited STAT3 to promote SPLUNC1 expression in M1 macrophages. **A** TRIM24 level in THP-1-derived Mφs treated with 15 ng/mL LPS + 20 ng/mL IFN-γ was determined by Western blotting. **B** Western blotting analysis of TRIM24 and SPLUNC1 levels in THP-1-derived Mφs after transfection with shTRIM24 or shNC for 48 h. **C** Flow cytometry for detection of M1 (CD86 + CD11b + ) macrophages in THP-1-derived Mφs transfected with shTRIM24 or shNC with or without treatment with 15 ng/mL LPS + 20 ng/mL IFN-γ. **D** Illustration of predicted binding sites of TRIM24 to SPLUNC1 promoter. **E** ChIP assay validated the binding of TRIM24 to a series of binding sites (BS 1-7) on SPLUNC1 promoter. **F** Dual luciferase reporter assay was performed to verify the interaction between TRIM24 and SPLUNC1 promoter. **G** Illustration of predicted binding sites of STAT3 to SPLUNC1 promoter. **H** The enrichment of BS 1-3 on SPLUNC1 promoter after immunoprecipitation by STAT3 antibody was assessed. **I** Dual luciferase reporter assay was conducted to validate the interplay between STAT3 and SPLUNC1 promoter. **J** The interaction between STAT3 and TRIM24 proteins in THP-1-derived Mφs was confirmed by Co-IP assay. **K** The enrichment of TRIM24 or STAT3 binding to SPLUNC1 promoter after TRIM24 down-regulation in THP-1-derived Mφs was validated by ChIP. **L** The promoter activity of SPLUNC1 in THP-1-derived Mφs after transfection with sh-TRIM24, sh-STAT3, or sh-TRIM24+sh-STAT3 was measured by dual-luciferase reporter assay. *p < 0.05, **p < 0.01, ***p < 0.001. For (**A**–**C**–**E**, **F**–**H**, **I**–**K**) Student’s *t*-test was performed. For B, L, one-way ANOVA followed by Tukey’s multiple comparison test was performed.

Given that TRIM24 was involved in the regulatory mechanism of macrophage polarization [11], and TRIM24 was predicted to possess binding sites in SPLUNC1 promoter, we further evaluated the involvement of TRIM24 in the modulation of SPLUNC1 during M1 macrophage polarization. First, an elevation of TRIM24 protein expression was observed in LPS/IFN-γ-induced M1 macrophages (Fig. 5A). Moreover, SPLUNC1 was down-regulated in TRIM24-depleted macrophages (Fig. 5B). Additionally, the enhanced percentage of M1 macrophages (CD11b^+^ CD86^+^ ) by LPS/IFN-γ was reduced after TRIM24 knockdown (Fig. 5C). Figure 5D illustrated the seven predicted binding sites (BS1-7) of TRIM24 in SPLUNC1 promoter. ChIP assay demonstrated that TRIM24 could bind to BS1, BS2, BS4 and BS5, among which BS5 presented the highest enrichment (Fig. 5E). In addition, inhibition of TRIM24 significantly repressed the luciferase activity of SPLUNC1 WT group, but not of SPLUNC1 MUT group (Fig. 5F). Therefore, TRIM24 could directly bind to SPLUNC1 promoter. Besides, the relative luciferase activity was not changed in SPLUNC1-silenced Mφs. However, LPS/IFN-γ treatment significantly enhanced the relative luciferase activity, which may be due to LPS/IFN-γ-induced TRIM24 up-regulation that promoted SPLUNC1 transcription (Supplementary Fig. 1E). On the other hand, STAT3 (BS1-3) (Fig. 5G), STAT1 (Supplementary Fig. 2A), and STAT6 (Supplementary Fig. 2B) were predicted to bind to SPLUNC1 promoter. ChIP assay confirmed that STAT3 directly bound to BS2 of SPLUNC1 promoter (Fig. 5H). Furthermore, the luciferase activity of SPLUNC1 WT group was lowered after STAT3, STAT1, or STAT6 silencing, whereas we did not observed changes in MUT group (Fig. 5I, Supplementary Fig. 2C, D). The direct interaction between TRIM24 and STAT3 protein was validated by Co-IP assay (Fig. 5J). However, TRIM24 protein could not interact with STAT1 or STAT6 protein (Supplementary Fig. 2E). Thus, STAT3 was selected in the subsequent experiments. Additionally, the binding of TRIM24 or STAT3 to SPLUNC1 promoter was attenuated by TRIM24 knockdown (Fig. 5K). Notably, the luciferase activity of SPLUNC1 WT group was reduced by TRIM24 or STAT3 knockdown, which was more evident when double knockdown of TRIM24 and STAT3 (Fig. 5L). In summary, TRIM24 facilitated M1 macrophage polarization by recruiting STAT3 to promote SPLUNC1 expression.

### USP7 stabilized TRIM24 via deubiquitination of TRIM24

**Fig. 6:**
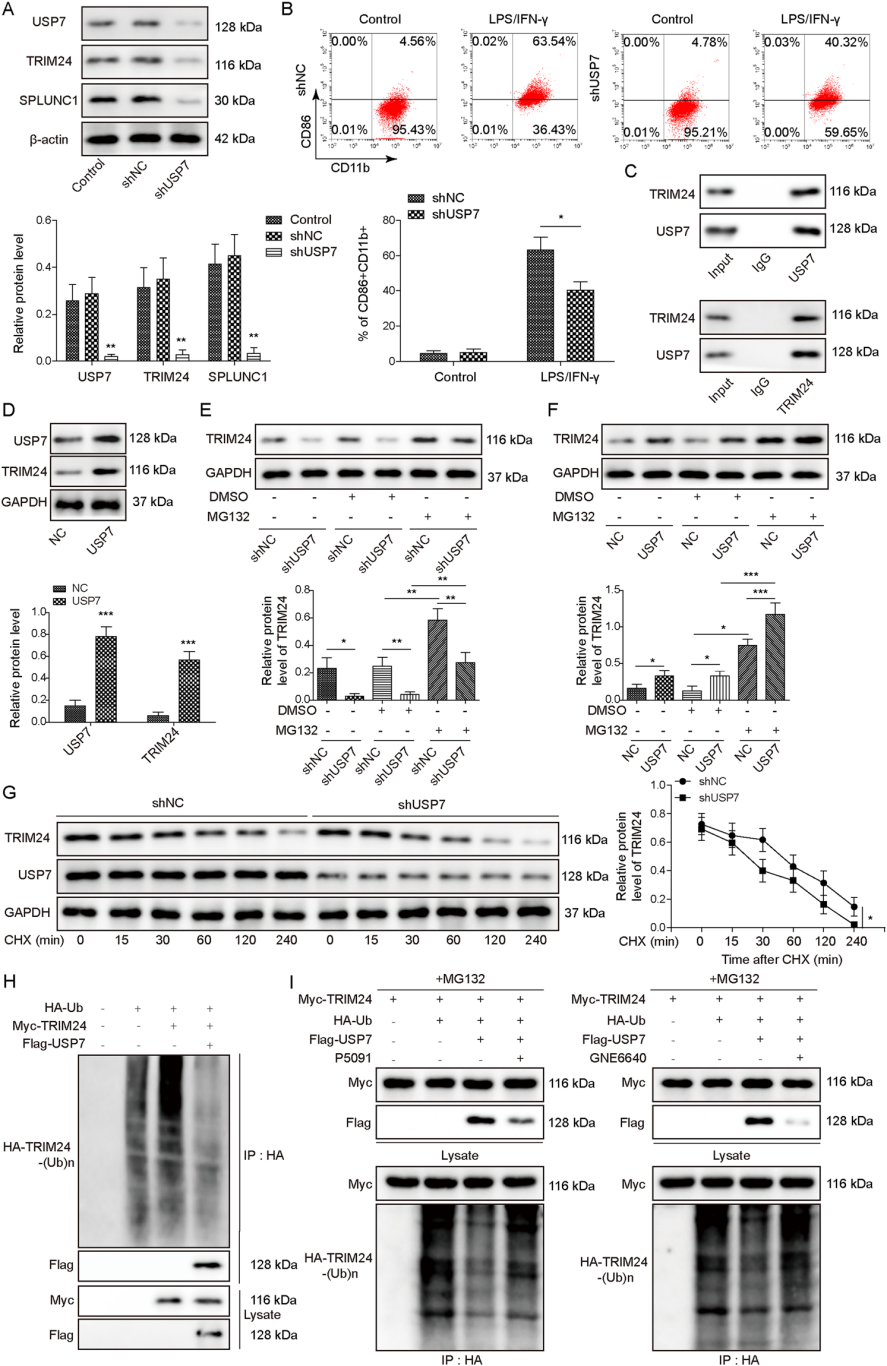
USP7 led to deubiquitination and stabilization of TRIM24. **A** Western blotting for detection of USP7, TRIM24, and SPLUNC1 levels in THP-1-derived Mφs after transfected with shUSP7 or shNC for 48 h. **B** M1 (CD86 + CD11b + ) macrophages in THP-1-derived Mφs transfected with shUSP7 or shNC was determined by flow cytometry. **C** Co-IP assay for confirming the protein interaction between USP7 and TRIM24 in THP-1-derived Mφs. **D** The protein expression of USP7 and TRIM24 in THP-1-derived Mφs after transfection with USP7 overexpression plasmid was assessed by Western blotting. **E**, **F** TRIM24 protein level in USP7-depleted or overexpressed THP-1-derived Mφs with or without MG132 treatment for 12 h was measured by Western blotting. **G** The degradation of TRIM24 protein in THP-1-derived Mφs after administration with CHX for 15, 30, 60, 120, 240 min was evaluated by Western blotting. **H** HEK293T cells were transfected with HA-Ub, Myc-TRIM24, or Flag-USP7 plasmids. The deubiquitination of USP7 on TRIM24 in HEK293T cells was detected by Co-IP assay. **I** HEK 293T cells were transfected with HA-Ub, Myc-TRIM24, or Flag-USP7 plasmids and treated with or without USP7 inhibitors P5091(4 μM) or GNE6640 (0.75 μM). The ubiquitination level of TRIM24 in THP-1-derived Mφs was evaluated by Co-IP assay. *p < 0.05, **p < 0.01, ***p < 0.001. For (**D**–**G**) Student’s *t*-test was performed. For (**A**, **B**–**E**, **F**) one-way ANOVA followed by Tukey’s multiple comparison test was performed.

To delineate the up-stream molecular mechanism of TRIM24, we were concerned about the deubiquitinating enzyme USP7 that was predicted to have deubiquitination sites in TRIM24. Down-regulation of USP7 restrained TRIM24 and SPLUNC1 expression in THP-1-derived Mφs (Fig. 6A). Accordingly, USP7 knockdown reduced LPS/IFN-γ- enhanced M1 macrophage percentage (Fig. 6B). The interplay between USP7 and TRIM24 was verified by Co-IP assay (Fig. 6C). Moreover, TRIM24 protein level was enhanced in USP7-overexpressed macrophages (Fig. 6D). To further verify whether TRIM24 degradation via ubiquitination pathway, THP-1-derived Mφs were treated with MG132 for 12 h, a proteasome inhibitor. We found that TRIM24 level was raised after treatment with MG132, which could be reduced by USP7 depletion, but further reinforced by USP7 overexpression (Fig. 6E, F). Furthermore, cycloheximide (CHX)-induced TRIM24 degradation was further enhanced after USP7 knockdown (Fig. 6G). More importantly, the ubiquitination of TRIM24 was weakened by USP7 (Fig. 6H). Moreover, USP7-mediated de-ubiquitination of TRIM24 was counteracted by treatment with USP7 inhibitors P5091 or GNE6640 (Fig. 6I). Therefore, USP7 could enhance TRIM24 expression in macrophages via de-ubiquitination of TRIM24.

### USP7 exerted anti-tumor effect by promoting M1 macrophage polarization via stabilization of TRIM24 to up-regulate SPLUNC1

**Fig. 7:**
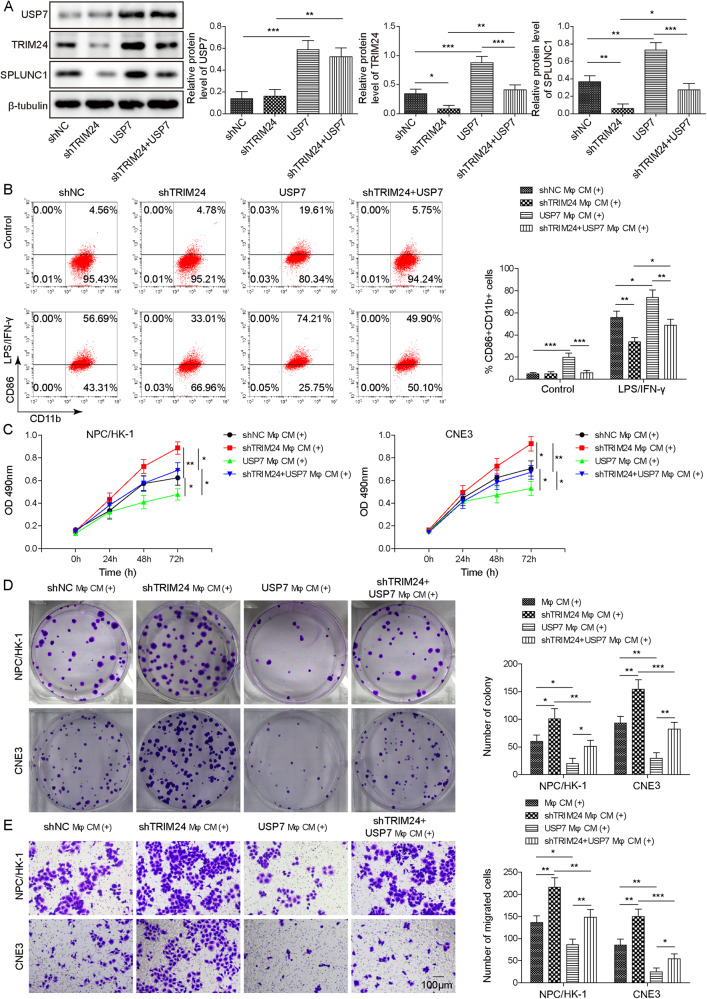
USP7 inhibited growth and metastasis of NPC cells by facilitating M1 macrophage polarization via TRIM24/SPLUNC1 axis. THP-1-derived Mφs were transfected with USP7 overexpression plasmid, shTRIM24, or a combination of them. **A** Protein abundance of USP7, TRIM24, and SPLUNC1 was evaluated by Western blotting. **B** Percentage of M1 (CD86^+^ CD11b^+^ ) in THP-1-derived Mφs was assessed by flow cytometry. NPC/NK-1 and CNE3 cells were treated with CM collected from THP-1-derived Mφs transfected with USP7 overexpression plasmid, shTRIM24, or USP7 overexpression plasmid+ shTRIM24. **C**, **D** CCK-8 and colony formation assay for evaluation of the growth of NPC/NK-1 and CNE3 cells treated with CM from different groups. **E** The migratory capacity of NPC cells (NPC/NK-1 and CNE3) was analyzed by Transwell assay. Scale bar = 100 μm. *p < 0.05, **p < 0.01, ***p < 0.001. One-way ANOVA followed by Tukey’s multiple comparison test was performed.

To further investigate the influence of USP7-mediated M1 macrophage polarization on the malignant capacities of NPC cells, USP7 overexpression plasmid, shTRIM24, or a combination of them was transfected into NPC cells. USP7 overexpression led to increase in TRIM24 and SPLUNC1 levels, which was counteracted by shTRIM24 transfection (Fig. 7A). Additionally, enforced expression of USP7 promoted M1 macrophage polarization, however, co-transfection with shTRIM24 abolished the promoting role of USP7 overexpression in the presence or absence of LPS/IFN-γ (Fig. 7B). Functional experiments further indicated that administration with CM from USP7-overexpressed macrophages suppressed growth and migration of NPC cells, which were abrogated by TRIM24 knockdown (Fig. 7C–E). Altogether, USP7 inhibited NPC progression by promoting M1 macrophage polarization via regulating TRIM24/SPLUNC1 axis.

### USP7 suppressed NPC proliferation and metastasis in vivo via TRIM24/SPLUNC1 axis-mediated M1 macrophage polarization

**Fig. 8:**
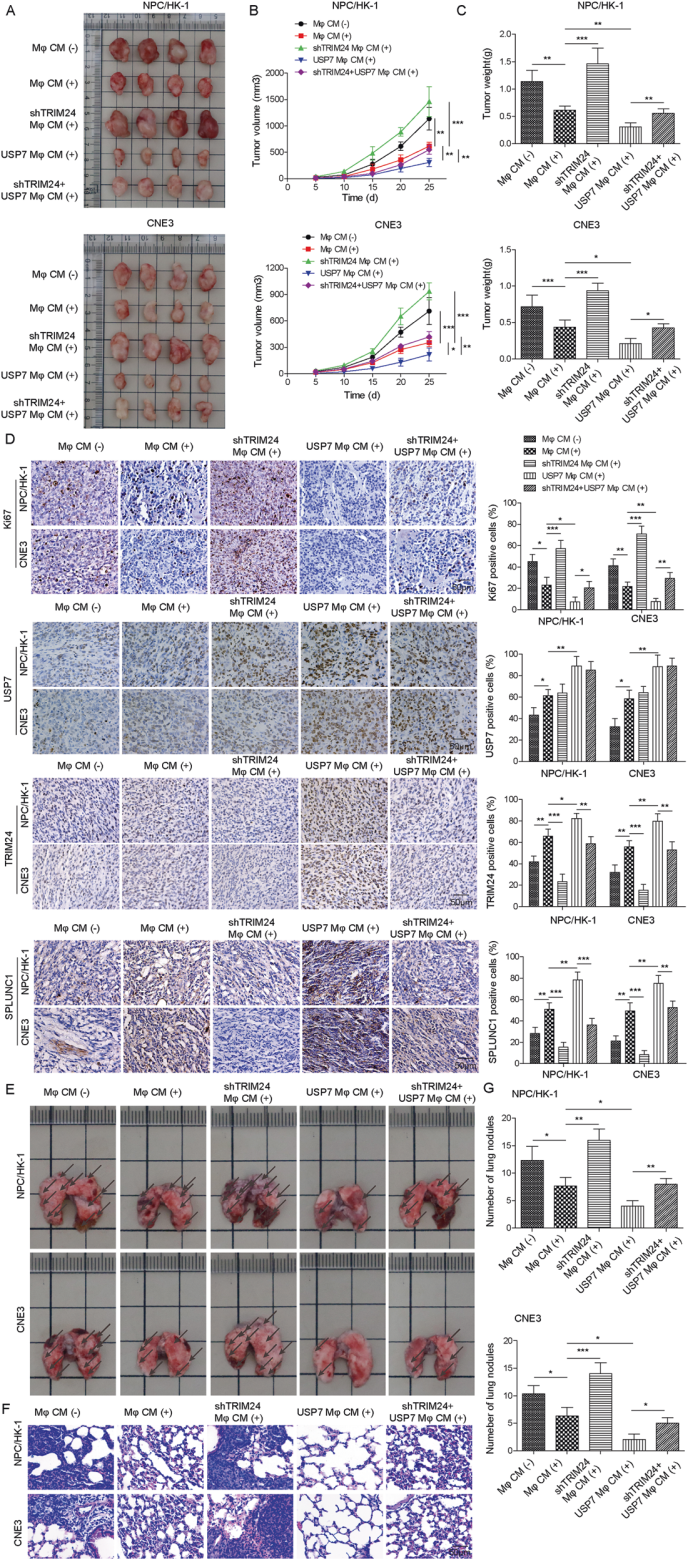
USP7 delayed NPC progression in vivo via promoting TRIM24/SPLUNC1 axis-mediated M1 macrophage polarization. Nude mice were subcutaneously injected with NPC/NK-1 and CNE3 cells that were suspended in PBS or CM from THP-1-derived Mφs transfected with shTRIM24, USP7 overexpression plasmid or shTRIM24 + USP7 overexpression plasmid. **A** Representative images of tumors, (**B**) tumor growth, (**C**) tumor weight in nude mice. **D** Immunohistochemical staining of Ki67, TRIM24, USP7 and SPLUNC1 expression in tumor sections. Scale bar = 50 μm. Nude mice were injection with NPC cells (NPC/NK-1 and CNE3) resuspended in PBS or CM from THP-1-derived Mφs with various transfections by tail vein. **E** Representative images of lungs of nude mice 2 months after injection. Scale bar = 1 cm. **F**, **G** HE staining for observing metastatic lesions in lung tissues and the numbers of metastatic lesions were quantified. Scale bar = 50 μm. *p < 0.05, **p < 0.01, ***p < 0.001. One-way ANOVA followed by Tukey’s multiple comparison test was performed.

Finally, we validated the above in vitro findings in nude mice in vivo. Treatment with CM from macrophages effectively reduced tumor volume and weight, which was more obvious when treatment with CM from USP7-overexpressed macrophages. Instead, depletion of TRIM24 in macrophages promoted tumor growth, and also reversed the inhibitory effect of USP7 overexpression (Fig. 8A–C). As assessed by IHC staining, Ki67 expression was lowered and SPLUNC1 expression was elevated after macrophage CM treatment, and CM-mediated down-regulation of Ki67 and up-regulation of SPLUNC1 could be reversed by TRIM24 knockdown, but promoted by USP7 overexpression. TRIM24 silencing also counteracted USP7 overexpression-mediated down-regulation of Ki67 and up-regulation of SPLUNC1 in tumors (Fig. 8D). The knockdown of TRIM24, SPLUNC1 and overexpression of USP7 were validated by IHC staining (Supplementary Fig. 3). Consistently, the number of metastatic lesions was diminished in lungs of mice treated with CM from macrophages. The anti-metastasis role of CM was enhanced by USP7 overexpression, but attenuated by TRIM24 down-regulation. USP7 overexpression-mediated inhibition in lung metastasis was neutralized by TRIM24 depletion (Fig. 8E–G). These data indicated that USP7 regulated TRIM24/SPLUNC1 axis to weaken the proliferation and metastatic capacities of NPC cells in vivo via promoting M1 macrophage polarization.

## Discussion

Accumulating evidence has clarified the vital roles of immune cells in the tumor microenvironment in orchestrating cancer development [20]. During malignant progression, tumor-associated macrophages may convert to an immunosuppressive phenotype to repress anti-cancer action of T cells [21]. Selective reprogramming of tumor-associated macrophages from immunosuppressive M2 to antineoplastic M1 phenotype represents an effective anti-tumor strategy [20]. Therefore, uncovering the mechanisms of macrophage phenotype transition in NPC microenvironment is conducive to delay NPC progression. In this work, we discovered that USP7 promoted macrophages transferred to antitumor M1 phenotypes, which inhibited NPC cell proliferation and metastasis. Mechanistically, USP7 deubiquitinated TRIM24 to stabilize TRIM24 expression, which subsequently increased SPLUNC1 expression via recruiting STAT3. Our findings indicated that USP7-induced M1 macrophage polarization may be a novel strategy for treating NPC.

SPLUNC1 is considered to act as a defensive protein in innate immunity system [8]. Recently, growing evidence has supported the crucial roles of SPLUNC1 in NPC development. For instance, SPLUNC1 was verified to be down-regulated in NPC biopsies and overexpression of SPLUNC1 suppressed NPC cell growth and induced apoptosis via miR-141-PTEN/p27 axis [22, 23]. Zhang et al. documented that SPLUNC1 contributed to all-trans-retinoic acid-mediated proliferation inhibition of NPC cells [10]. In this study, we found that single LPS stimulation did not promote CD86 and SPLUNC1 expression, whereas LPS exerted promotive effect on IFN-γ-mediated up-regulation of CD86 and SPLUNC1. Therefore, LPS + IFN-γ treatment was used to induce M1 macrophage polarization. We provided first evidence that SPLUNC1 was up-regulated during M1 macrophage polarization. Further experiments revealed that SPLUNC1 was responsible for M1 macrophage polarization, which restrained growth and migration of NPC cells. However, our results did not show the other metastatic phenotypes of NPC cells, such as invasion, which needs to be explored in the future. The activation of JAK/STATs signaling pathway has been demonstrated after IFN-γ treatment. JAK2 is a critical regulator of macrophage function and LPS/IFN-γ stimulation leads to phosphorylation of JAK2 and subsequent STAT1 phosphorylation in macrophages [24]. Inactivation of JAK/STATs pathway has been documented to repress M1 macrophage polarization [25]. In line with these findings, we demonstrated that JAK/STATs pathway activation was responsible for SPLUNC1-mediated M1 macrophage polarization. These findings suggested that SPLUNC1 delayed NPC progression via inducing M1 macrophage polarization via activating JAK/STATs pathway.

Subsequently, we found that TRIM24 functioned as an upstream regulator of SPLUNC1 in macrophages. TRIM24 is a member of TRIM family proteins, which exerts emerging roles in the progression of diverse malignances [26–28]. Notably, TRIM24 down-regulation was documented to drive macrophage M2 polarization via repressing STAT6 acetylation [11]. Herein, we firstly reported that TRIM24 was up-regulated in M1 macrophages, which recruited STAT3 to enhance SPLUNC1 expression. Notably, our results indicated that SPLUNC1 promoted STAT3 activation, which may further contribute to SPLUNC1 transcription. Therefore, there was a positive feedback loop between SPLUNC1 and STAT3. To sum up, TRIM24 initiated SPLUNC1-STAT3 feedback loop to contribute to M1 macrophage polarization.

Deubiquitination and ubiquitination have been considered as two ways of protein post‐translational regulation to maintain cellular protein homeostasis during various pathophysiological processes [29]. Dysregulation of deubiquitination or ubiquitination occurs in a series of disorders, especially in malignancy [30]. USP7, as one of members of deubiquitinating enzymes, participates in the development of multiple tumors. For example, USP7 delayed renal cell carcinoma progression via deubiquitylating and stabilizing ARMC5 [14]. Wang et al. documented that USP7-mediated Tip60 stabilization contributed to inflammation in acute lung injury [31]. Interestingly, in this study, USP7 was predicted as a TRIM24‐associated deubiquitinase. Meantime, our results provided first evidence that USP7 stabilized TRIM24 via direct deubiquitylation, which restrained NPC proliferation and metastasis by promoting M1 macrophage polarization via up-regulating SPLUNC1.

**Fig. 9:**
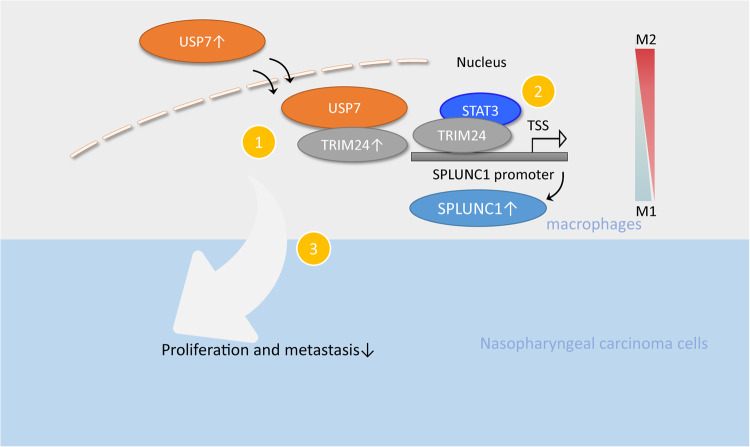
Schematic diagram of this study. USP7 promotes the polarization of macrophages to M1 phenotype through de-ubiquitination and stabilization of TRIM24 that recruits STAT3 to trigger SPLUNC1 transcription, thereby repressing NPC cell growth and metastasis.

In conclusion, our observations revealed that USP7 reprogrammed macrophages to M1 phenotype by deubiquitination and stabilization of TRIM24 to enhance SPLUNC1 expression, and finally inhibited NPC growth and metastasis (Fig. 9). Our study suggests that targeting USP7/TRIM24/SPLUNC1 cascade might suppress the immunosuppressive effect and represent an effective immunotherapy strategy for NPC. Our findings greatly contribute to our understanding of the function and mechanisms of USP7 in the modulation of NPC progression. Therefore, our observations provide a novel therapeutic idea for the treatment of NPC, which can be expected to greatly improve patients’ prognosis.

### Reporting summary

Further information on research design is available in the Nature Research Reporting Summary linked to this article.

### Supplementary information

Original Data File

Supplementary Materials

Reporting Summary

## Data Availability

The Original western blot band were shown in URL: https://osf.io/kb4sp/; 10.17605/OSF.IO/KB4SP.

## Materials availability

The original western blot band were shown in URL: https://osf.io/kb4sp/; 10.17605/OSF.IO/KB4SP.
