# Supplementary figures and images for: USP7 inhibits the progression of nasopharyngeal carcinoma via promoting SPLUNC1-mediated M1 macrophage polarization through TRIM24
Source: Cell Death Dis. 2023 Dec 21;14(12):852. doi: 10.1038/s41419-023-06368-w (PMC10739934; doi:10.1038/s41419-023-06368-w)

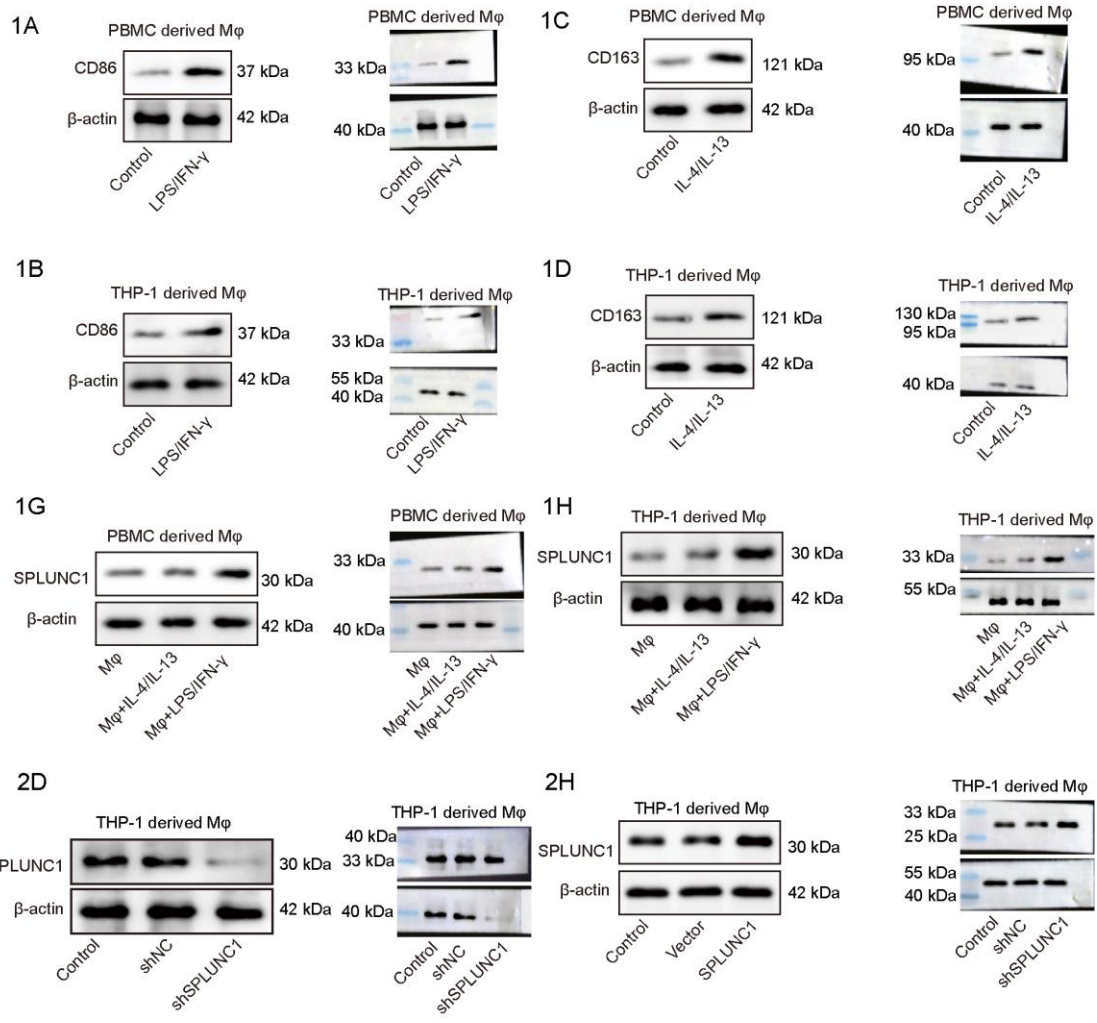

3A

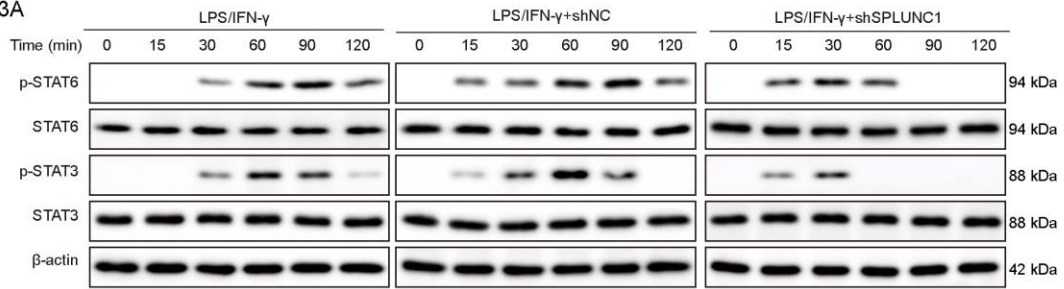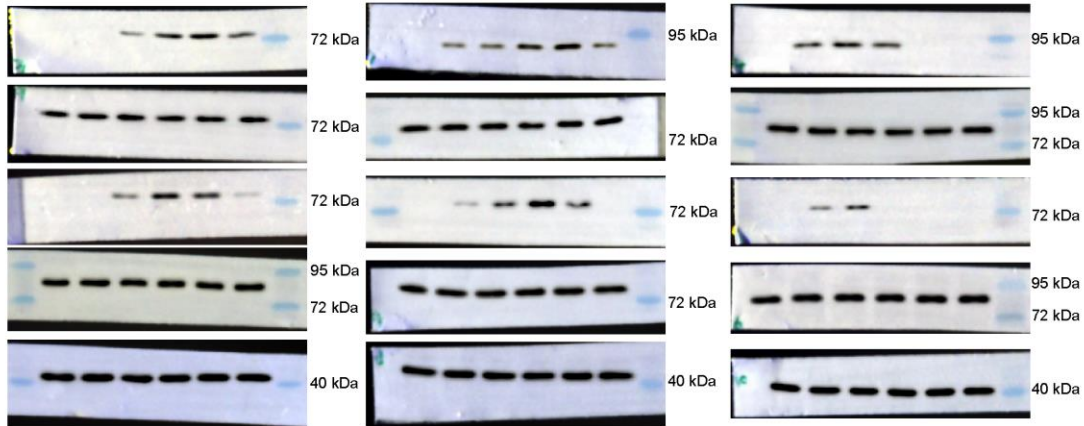

3B

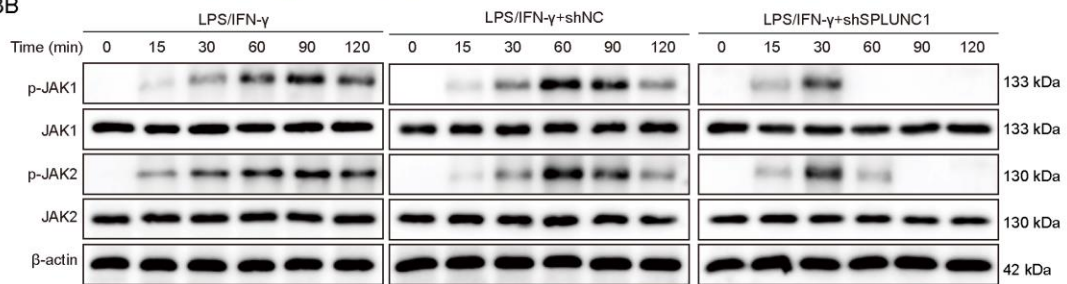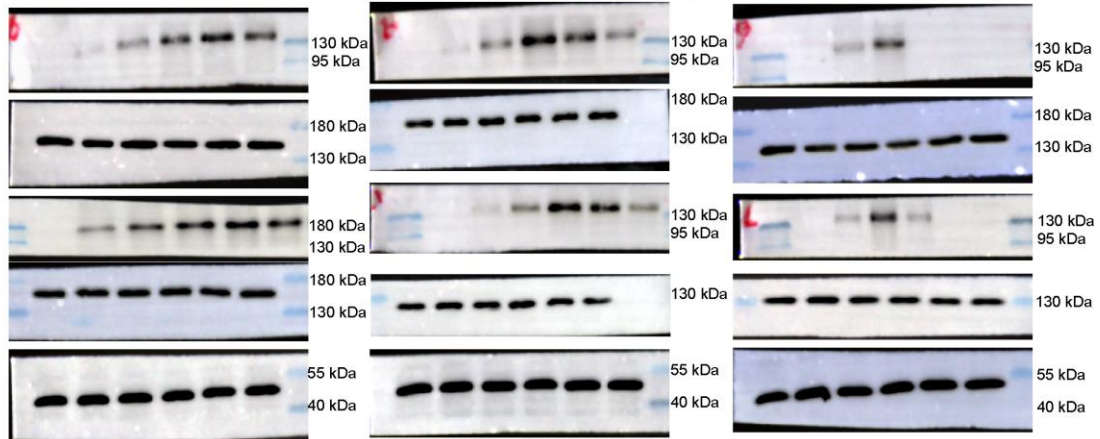

3D

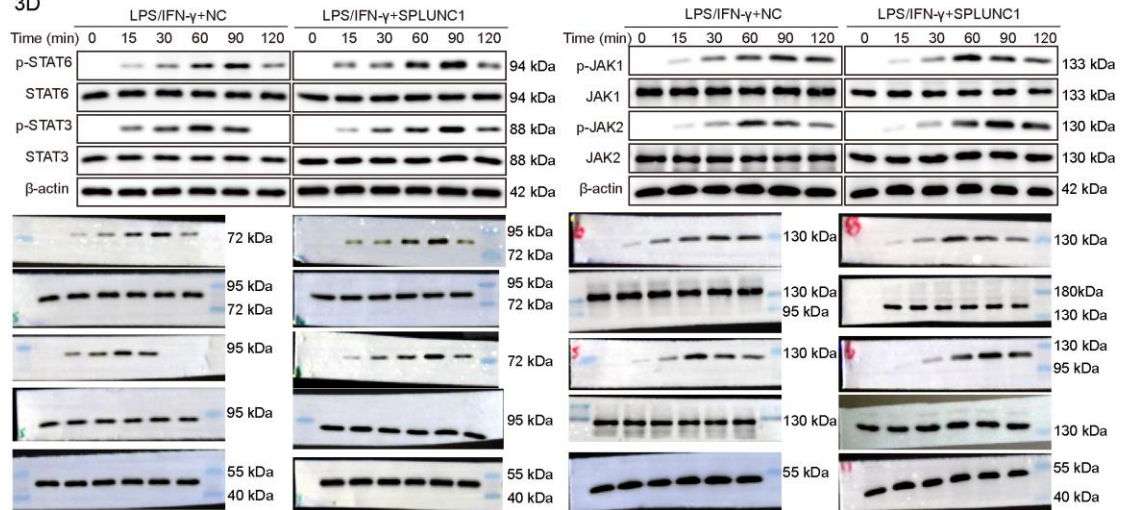

5A

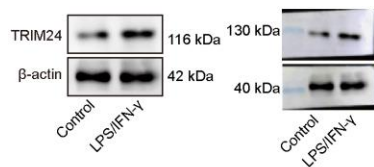

5B

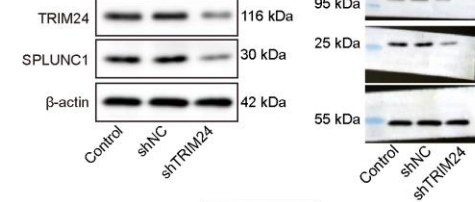

5J

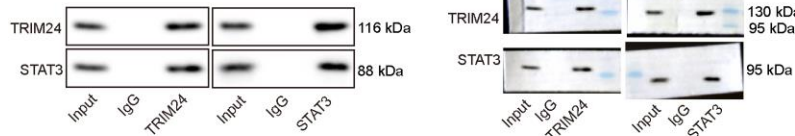

6A

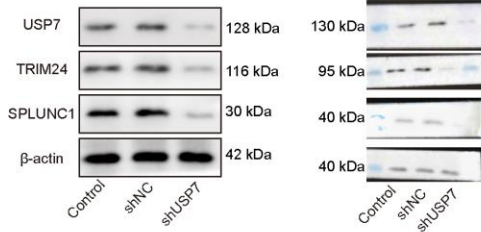

6C

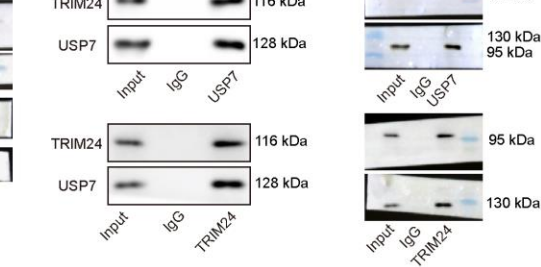

6D

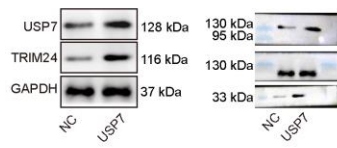

6E

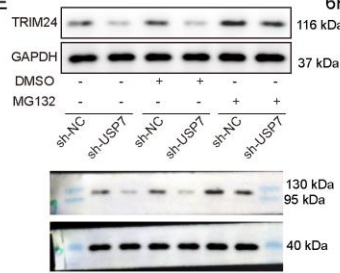

6F

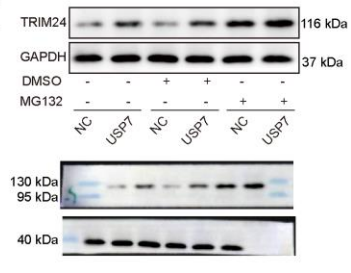

6G

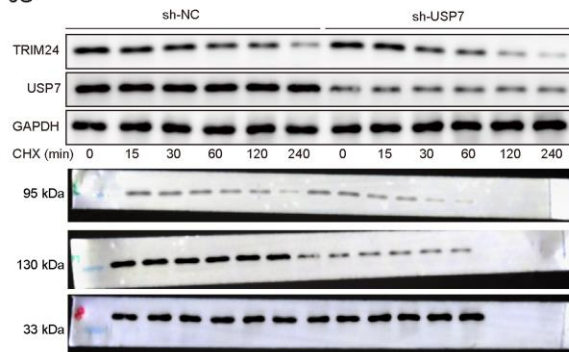

6H

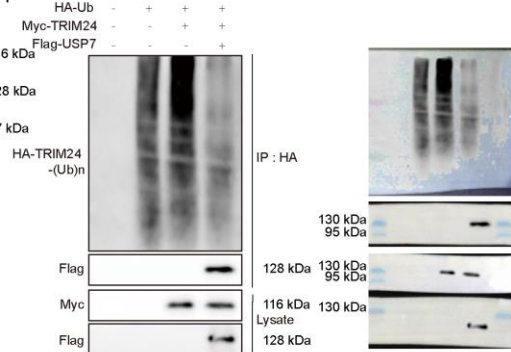

6I

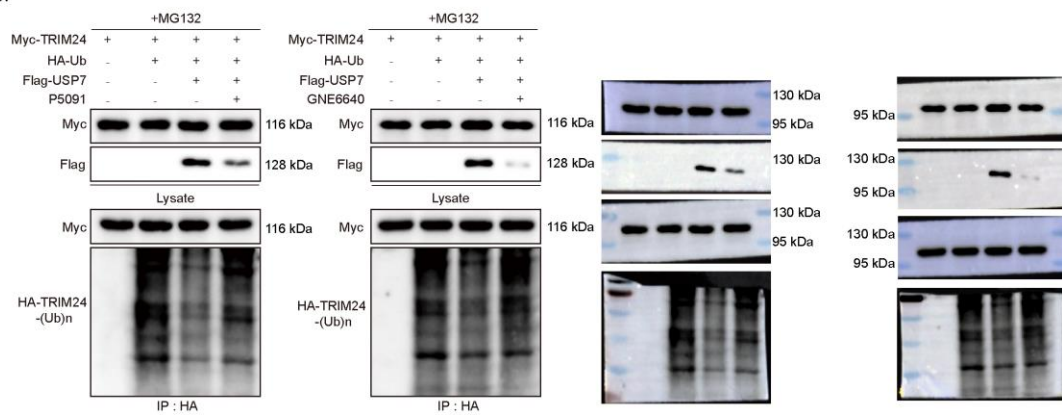

7A

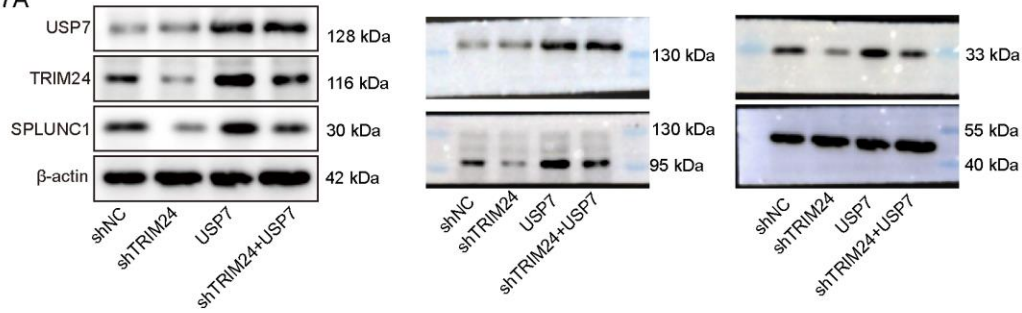

S1A

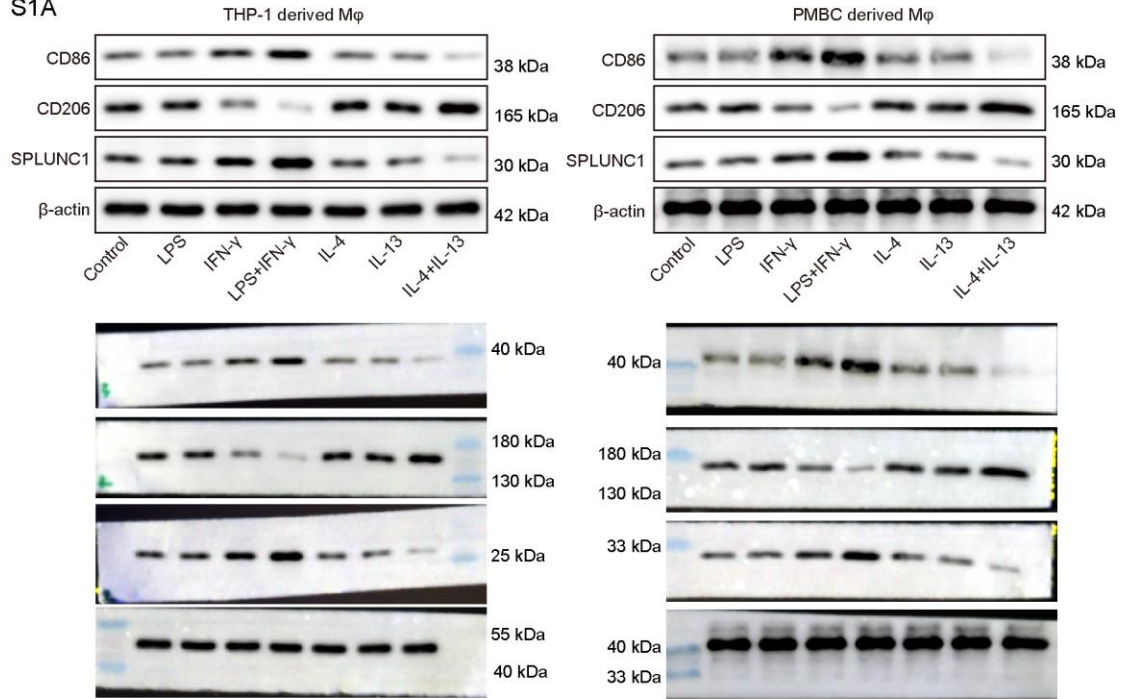

S1B

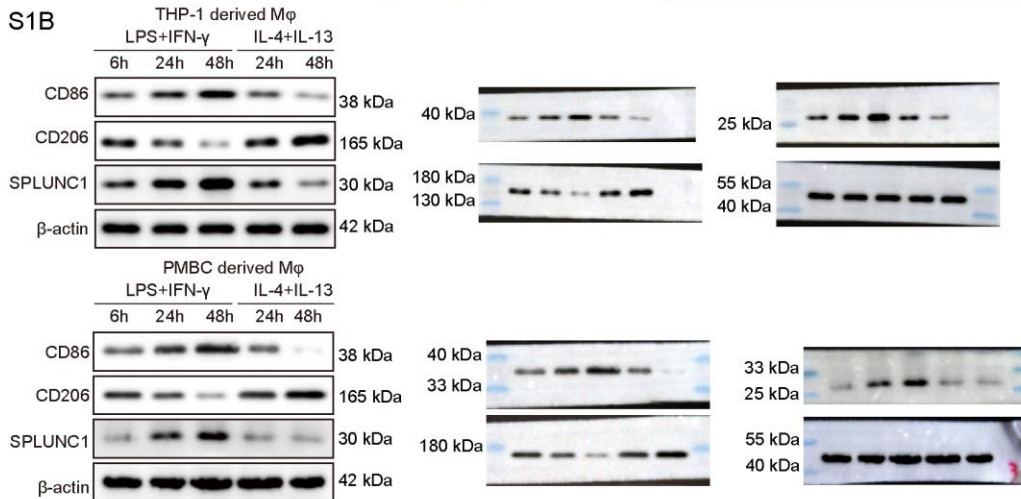

S2E

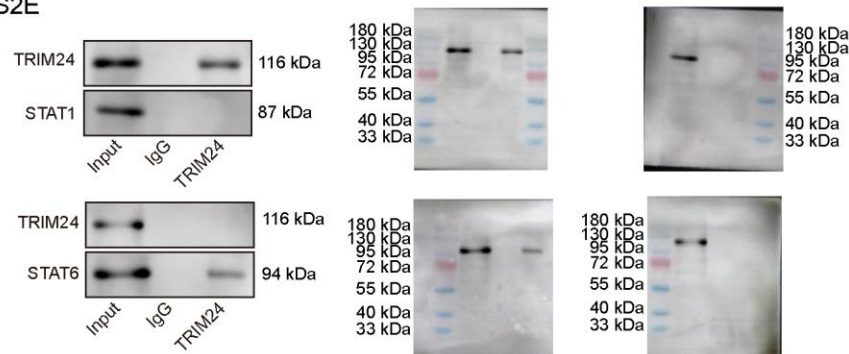

Supplement: Supplementary file 1 — Original Data File [file 41419_2023_6368_MOESM1_ESM.pdf]
