# Supplementary material for: USP7 inhibits the progression of nasopharyngeal carcinoma via promoting SPLUNC1-mediated M1 macrophage polarization through TRIM24
Source: Cell Death Dis. 2023 Dec 21;14(12):852. doi: 10.1038/s41419-023-06368-w (PMC10739934; doi:10.1038/s41419-023-06368-w)
Supplement: Supplementary file 2 — Supplementary Materials [file 41419_2023_6368_MOESM2_ESM.pdf]

Supplementary Materials

Supplementary Figure and Figure legend

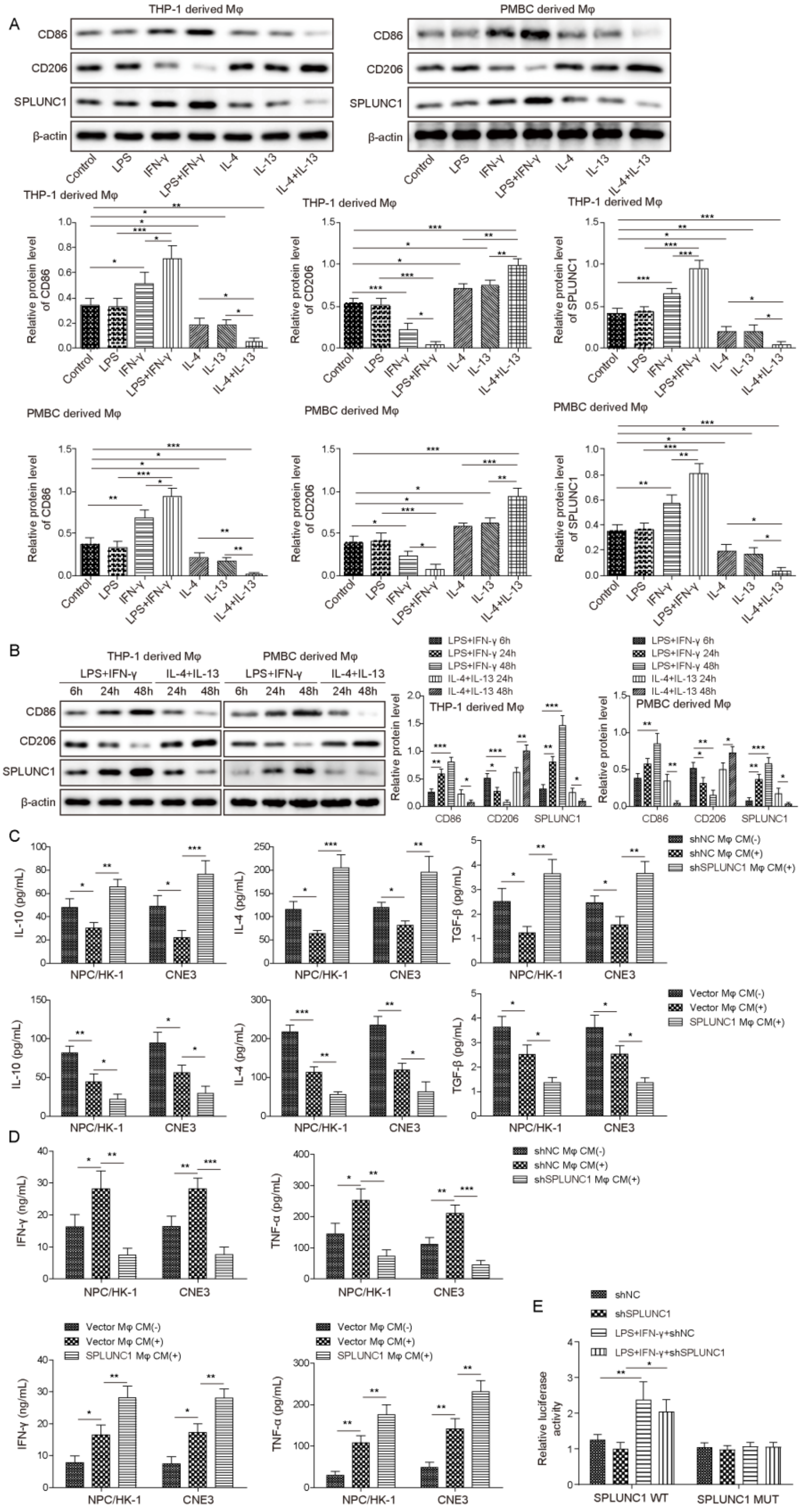

Supplementary Figure 1. (A) PBMC- or THP-1-derived Mφs were stimulated by 15 ng/mL LPS, 20 ng/mL IFN-γ, 15 ng/mL LPS+20 ng/mL IFN-γ, 20 ng/mL IL-4, 20 ng/mL IL-13, or 20 ng/mL IL-4+20 ng/mL IL-13 for 48 h. SPLUNC1, CD86, and CD206 protein levels in PBMC- or THP-1-derived Mφs were detected by Western blotting. (B) PBMC- or THP-1-derived Mφs were stimulated with 15 ng/mL LPS+20 ng/mL IFN-γ for 6, 24, 48 h; or treated with 20 ng/mL IL-4+20 ng/mL IL-13 for 24, 48 h. SPLUNC1, CD86, and CD206 protein levels in PBMC- or THP-1-derived Mφs were evaluated by Western blotting. (C-D) NPC cells were administrated with CM collected from THP-1-derived Mφs transfected with shNC, shSPLUNC1, vector, or SPLUNC1 overexpression plasmid. The release of IL-10, IL-4, TGF-β, TNF-γ, and TNF-α from NPC cells was determined by ELISA. (E) THP-1-derived Mφs with or without LPS+ IFN-γ treatment were transfected with shSPLUNC1 or shNC in combination with SPLUNC1 luciferase reporter plasmid. The transcription activity of SPLUNC1 was measured by dual luciferase reporter assay. *$p < 0.05$, **$p < 0.01$, ***$p < 0.001$. One-way ANOVA followed by Tukey's multiple comparison test was performed.

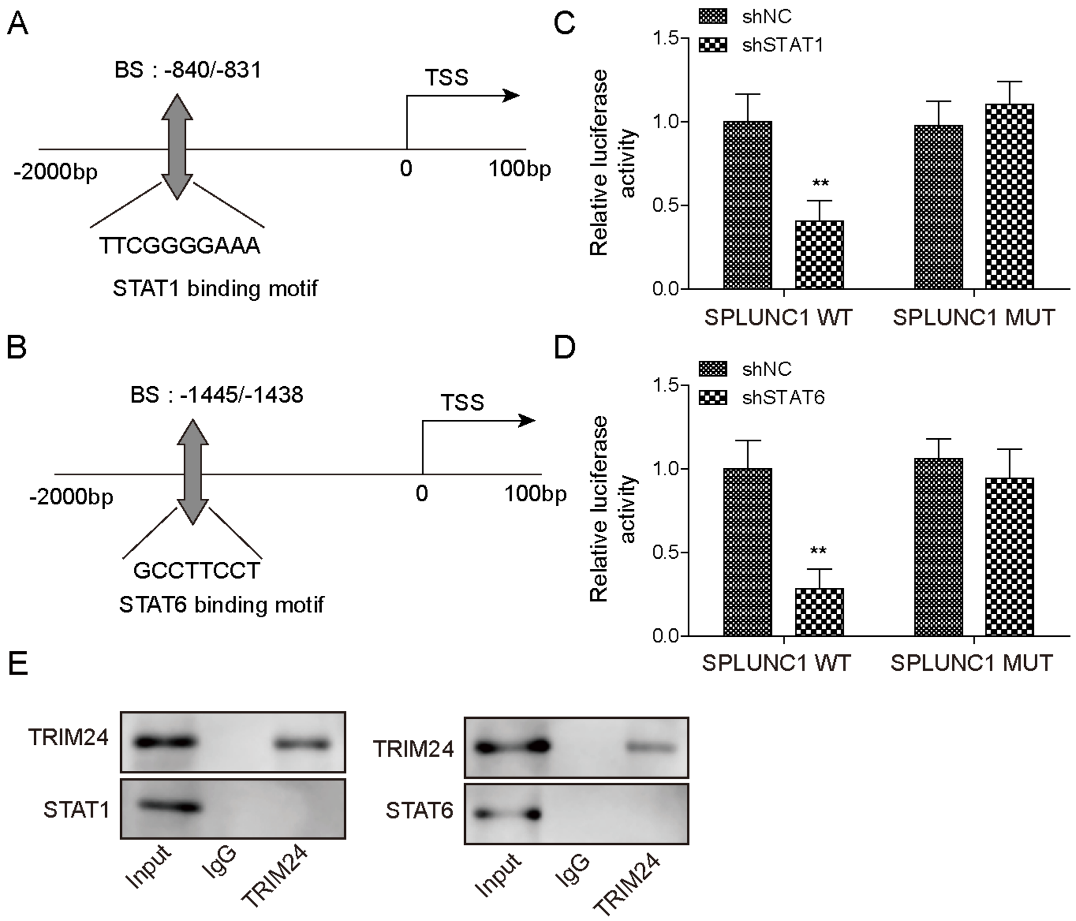

Supplementary Figure 2. (A) Illustration of predicted binding sites of STAT1 to SPLUNC1 promoter. (B) Illustration of predicted binding sites of STAT6 to SPLUNC1 promoter. (C) The interaction between STAT1 and SPLUNC1 promoter was verified by dual luciferase reporter assay. (D) The direct binding of STAT6 to SPLUNC1 promoter was evaluated by dual luciferase reporter assay. (E) Co-IP assay determined the interaction between STAT1/6 and TRIM24 proteins. **p < 0.01. Student's t test was performed.

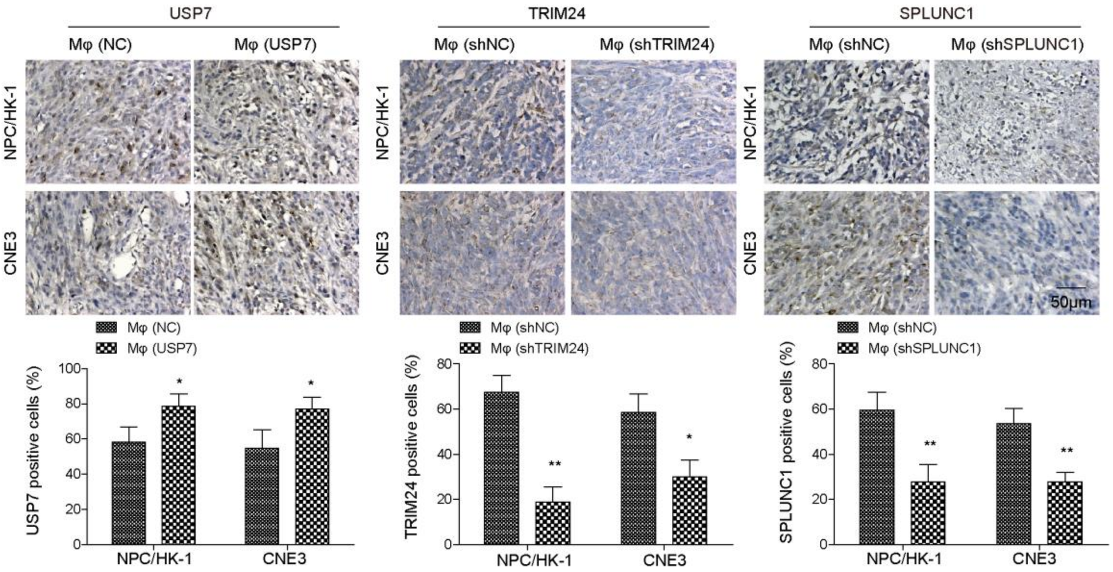

Supplementary Figure 3. Immunohistochemical staining detected the expression of TRIM24, USP7 and SPLUNC1 in tumor sections. Scale bar = 50 μm. $*p < 0.05$, $**p < 0.01$. Student's t test was performed.
